# Enhanced interfacial solar desalination using nano-engineered MoO_*x*_ photothermal evaporators†

**DOI:** 10.1039/d5na00249d

**Published:** 2025-05-19

**Authors:** Asghar Ali, Muhammad Qasim, Piotr A. Piatkowski, Ganjaboy Boltaev, Andra N. K. Reddy, Ali S. Alnaser

**Affiliations:** a Materials Science and Engineering Program, College of Arts and Sciences, American University of Sharjah Sharjah 26666 United Arab Emirates aalnaser@aus.edu mqasim@aus.edu; b Department of Physics, American University of Sharjah Sharjah 26666 United Arab Emirates; c Department of Chemical and Biological Engineering, American University of Sharjah Sharjah 26666 United Arab Emirates; d Materials Research Center, American University of Sharjah Sharjah 26666 United Arab Emirates

## Abstract

Interfacial solar desalination relies on enhanced optical absorbance, heat localization at the air/water interface, and effective water management on photothermal evaporators. However, its commercialization is hindered by marginal vaporization rates, processing challenges, and unacceptable stability. This study presents a novel substoichiometric molybdenum oxide (MoO_*x*_) solar absorber with a unique nanochannel-on-microchannel architecture, designed to enhance broadband absorbance, concentrate heat within thin layers of water, and promote superwicking. For the first time, a tightly focused, non-diffracting Bessel laser beam is employed to create nanochannels layered over hierarchically designed open microchannels. The nanochannels promote cluster evaporation by distributing water in very thin layers, while the hierarchical morphology and rough oxide microchannels contribute to strong broadband absorbance and generate capillary forces that enable superwicking on the surfaces at any angle. Outdoor tests demonstrated exceptional performance, with evaporation rates of 4.21 kg m^−2^ h^−1^ under 1 sun and 19.3 kg m^−2^ h^−1^ under 3 suns, outperforming existing evaporators. Comparison of these rates with indoor rates under controlled lab conditions suggests that ∼50% of the total evaporation rate was contributed by wind and ambient temperature. Moreover, the impact of water salinity on interfacial evaporation is revealed by performing experiments and comparing results from both saline and deionized water. Salt ions that are specifically adsorbed at the solution/MoO_*x*_ interface are found to inhibit direct contact between MoO_*x*_ and the secondary water, thereby enhancing evaporation by lowering the adsorption energy. A comprehensive analysis of hydrogen bonding states, the electrical double layer, temperature measurements, vaporization enthalpy, and efficiency calculations corroborates the performance improvements. Our findings demonstrate significant potential for large-scale solar desalination and provide new possibilities for advancing interfacial solar desalination.

## Abstract 2

Molybdenum oxide solar absorber, fabricated using Bessel laser beam, exhibits outstanding performance in interfacial solar desalination, offering a promising and sustainable solution for clean water production.

## Graphical abstract

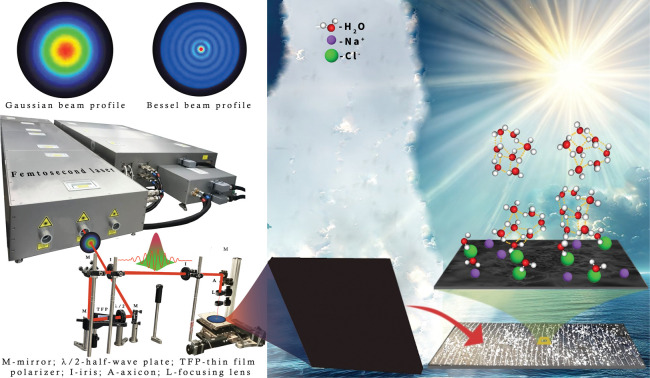

## 1. Introduction

Clean water is a highly scarce resource due to population growth, rapid industrialization, urbanization, and overuse and pollution of freshwater sources,^1,2^ with over 700 million people currently experiencing severe water shortages.^3^ Projections suggest that by 2050, nearly 7 billion people in 60 countries will face water scarcity.^4^ To address this, reverse osmosis (RO) desalination has become the primary source of global clean water. Despite its commercial success, RO remains challenged by its high energy consumption (∼2.5–4.0 kW h m^−3^),^5^ prompting research efforts for more efficient alternatives. One promising method to reduce desalination costs is solar-thermal desalination that harnesses solar heat to evaporate water, leaving salts behind, and then condenses the vapor into fresh water.^6^ Among the various solar–thermal desalination approaches, interfacial solar evaporation has recently garnered significant research interest due to its high efficiency.^7–9^ In this method, a photothermal evaporator is positioned at the liquid's surface, concentrating heat at the air–water interface.^10^ This design minimizes bulk water heating and only allows the surface layer to absorb the solar energy. As evaporation occurs, water is continuously drawn into the evaporator through specialized channels or pores. For optimal performance, the solar absorber must exhibit near-complete optical absorption across the entire solar spectrum (250–2500 nm), high light-to-heat conversion efficiency, superwicking properties, and low thermal conductivity.^9,10^ Additionally, it should be chemically stable, require minimal maintenance, support solar tracking and concentration, and be cost-effective.^11^

A variety of photothermal materials are used in interfacial solar evaporation,^12^ including carbon-based materials,^13–15^ noble metals,^16–18^ nanoparticles,^19^ semiconductors,^20^ organic coatings,^21^ and polymers.^22^ These are generally categorized based on the photothermal conversion mechanism: thermal vibration of molecules, non-radiative relaxation of semiconductors, and localized surface plasmon resonance (LSPR).^8,10,23^ Carbon-based materials convert solar energy to heat through thermal vibrations of carbon molecules, offering advantages such as low cost, availability, and scalability.^24–26^ However, they may suffer from low mechanical strength and difficulties in achieving stable powder deposition on substrates.^9^ Examples of low cost carbon-based photothermal materials include carbon monoliths from carbonized waste polyethylene terephthalate (PET) bottles,^24^ carbon black,^27,28^ and carbonized natural materials.^25,26,29^ Structurally stronger carbon materials such as nanotubes,^15^ graphene,^14^ and their derivatives,^13^ offer improved strength but involve complex processing and often reduced wettability. Semiconductor absorbers such as CuS,^30^ NiO,^31^ SnSe,^32^ and MoS_2_^33^ convert solar light to heat through electron–hole pair creation and their subsequent non-radiative relaxation. These typically require narrow band gaps for efficient conversion, which are often difficult to synthesize and may degrade under intense light and heat.^34^

Solar evaporators utilizing the LSPR effect are based on metallic nanoparticles that, when exposed to light at their resonance wavelengths, generate hot electrons that dissipate energy non-radiatively to increase the surface temperature.^8,10,35^ Typically, the plasmonic solar evaporators based on individual nanoparticles are narrowband, requiring the use of nanoparticles of a wider size distribution to allow for broadband absorption.^36^ Also, chemical stability and particle agglomeration remain challenges. In this regard, ultrafast lasers can play a significant role in fabricating efficient solar evaporators. These lasers can structure surfaces by delivering very high peak powers instantaneously, quickly ablating the surface and forming hierarchical morphologies with varying size features.^37,38^ This process imparts broadband light absorption to the laser-treated metal surface through the combined LSPR effects of particles of different sizes. Additionally, laser structuring can simultaneously bestow superhydrophilic and superwicking properties by surface oxidation during structuring in an oxygenated environment,^39,40^ which are essential for efficient water transport during interfacial solar evaporation. Only few studies have explored the use of laser-treated metals for this purpose. Yin *et al.*^41^ used femtosecond lasers on titanium (Ti) foam, achieving broadband solar absorption of >97% and an evaporation rate of 1.79 kg m^−2^ h^−1^ under 1 sun irradiance. However, the proposed evaporator design was not solar-trackable since it employed a floating evaporator that must always float on the water surface. Also, the porous solar evaporator was inherently susceptible to clogging when used with saline water feeds. The cost of Ti foam poses another limitation. Chen *et al.*^42^ used a picosecond laser to inscribe microchannels on the surface of an aluminum (Al) sheet. An optical absorbance of 47–85% was achieved in the VIS-NIR region and a modest evaporation rate of 1.24 kg m^−2^ h^−1^ was reported under 1 sun illumination, highlighting the need for improved and optimized laser structured solar evaporators. Another study by Singh *et al.*^11^ demonstrated a femtosecond laser-structured Al sheet in a similar vertical configuration for solar absorption. A broadband optical absorbance of 97% and an evaporation rate of 0.9 kg m^−2^ h^−1^ under 1 sun illumination were achieved. Under 3-sun irradiation, the highest reported evaporation rate was approximately 5 kg m^−2^ h^−1^. However, this study did not assess high-salinity or outdoor performance with natural sunlight. While these studies highlight the promise of laser-treated solar evaporators in boosting interfacial evaporation, further research is needed to boost the performance further to levels suitable for industrial deployment. Moreover, comprehensive evaluation under real-world conditions—such as outdoor environments—and systematic investigation of critical factors like salinity, wind, and concentrated solar irradiation are essential for practical implementation.

In this study, we fabricate a novel and highly-efficient molybdenum (Mo) oxide-based solar absorber for interfacial solar evaporation consisting of engineered nanochannels on top of hierarchically structured microchannels. Mo was chosen for its broadband optical absorbance and excellent photothermal conversion efficiency in both metallic and oxidized states,^43,44^ structural robustness,^45^ excellent ambient stability,^46^ thermal and chemical stability of its oxides,^47^ more localized laser ablation due to its refractory nature,^48,49^ and its role as an essential micronutrient.^50^ In addition, MoO_*x*_ structures exhibit superhydrophilicity which is essential for high wettability and water transport during interfacial solar evaporation. Owing to these favorable attributes, MoO_*x*_-based solar absorbers and evaporators have been previously explored; however, their performance has generally remained sub-optimal. For instance, Wang and co-workers^44^ developed a solar evaporator based on Mo nanoparticles embedded within an amorphous MoO_*x*_ matrix (Mo@MoO_*x*_). Although the reported solar absorptivity was 91%, the solar evaporator was not employed in solar evaporation. In another study, Wang and co-workers^51^ fabricated nano-multilayered MoO_*x*_-based solar evaporator coatings (SSACs) with a solar absorptivity of 93%, without evaluating its interfacial solar evaporation. Also, Wang and co-workers^52^ fabricated bi-layered MoO_*x*_ cermet-based SSACs. Although the solar absorptivity was 91%, the interfacial evaporation performance was not reported. A study by Lu *et al.*^53^ reported a MoO_*x*_-based solar evaporator, developing an oxygen-deficient MoO_*x*_-based evaporator in the form of ultrathin nanosheets loaded onto a PTFE membrane. The reported evaporation rate with seawater was 1.26 kg m^−2^ h^−1^ under 1 sun, which is lower than that of the MoO_*x*_-based evaporator reported in this study. Also, the MoO_*x*_/PTFE membrane proposed by Lu *et al.*^53^ was prepared by an extraction filtration method and the MoO_*x*_ layer is perceived to exhibit limited physical stability. While solar evaporators based on MoO_*x*_ have been reported, their performance in interfacial solar evaporation remains largely underexplored. As demonstrated in this study, judicious engineering of the MoO_*x*_ absorber holds significant promise for substantially increasing the evaporation rate.

For the first time, we demonstrate the use of a tightly focused, non-diffracting femtosecond laser Bessel beam to inscribe substoichiometric MoO_*x*_ nanochannels, uniquely integrated with open microchannels, for significant enhancements in interfacial solar evaporation. Compared to the commonly-used Gaussian beams, non-diffracting Bessel beams are more intense and tightly focusable, allowing for more precise, and narrower channels with fewer ablation effects on the surrounding material. To show the effect of morphology on interfacial evaporation, we compare the open microchannel morphology developed with a Gaussian beam to the nanochannel-superimposed microchannel morphology created by the Bessel beam. To illustrate the role of Mo delocalized d-orbital electrons in enhancing interfacial evaporation, we compare amorphous Al oxide (AlO_*x*_) evaporators produced with Bessel and Gaussian beams to the MoO_*x*_ evaporators. Moreover, the impact of water salinity on interfacial evaporation is revealed by performing experiments and comparing results from both saline and deionized (DI) water. To show that interfacial evaporation under concentrated solar irradiation favors MoO_*x*_ over AlO_*x*_, desalination experiments were performed under 1, 2, and 3 suns irradiance. Furthermore, Raman analysis of hydrogen bonding provides valuable information about changes in cluster evaporation in response to the electronic nature of the evaporator, its morphology, and adsorbed salt ions. Equilibrium potentials and Tafel plots provided insights into how salinity influences the evaporation rate by modifying the electrical double layer at the evaporator/water interface during interfacial evaporation. Finally, we show results from our outdoor experiments under real field conditions to demonstrate compatibility with solar tracking and concentrated solar irradiance.

## 2. Experimental

### 2.1. Fabrication of solar evaporators

**Fig. 1:**
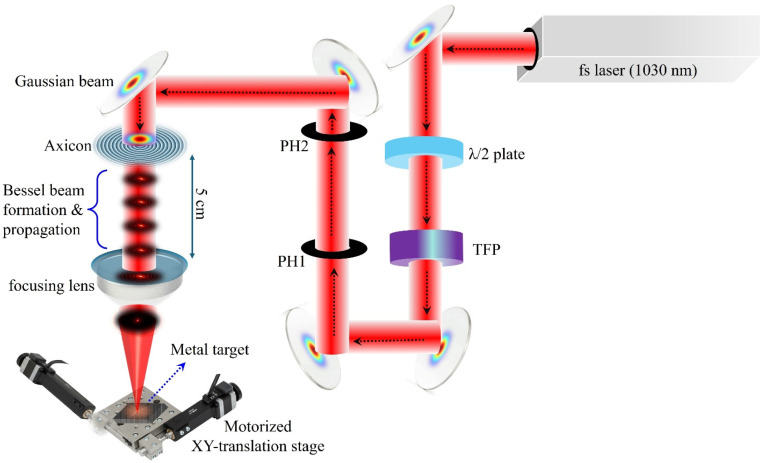
The schematic illustrating the surface processing procedure of metals using linearly polarised fs-Gaussian and Bessel laser beams. The setup includes fs-laser (fiber-based femtosecond laser); *λ*/2 plate – a half-wave plate for tuning the polarization state and power of the input beam; TFP – thin-film plate polarizer mounted at Brewster's angle that reflects the s-polarization and transmits the p-polarization of an input laser beam; PH1 & PH2 – a two pinhole arrangement for aligning the input laser beam that illuminates the axicon; and a lens (*f* = 5 cm) is employed to finely focus the laser beam onto the metal target mounted on a motorized XY-translation stage.

The solar evaporators were prepared by femtosecond laser structuring of Al and Mo samples (30 × 10 × 1 mm^3^) in air employing Gaussian and Bessel (also referred to as Bessel–Gauss) beams. A schematic of the experimental setup is shown in Fig. 1. The femtosecond (fs) laser source is a fiber-based laser (AFSUFFL-300-2000-1030-300, Active Fiber Systems GmbH, Germany) with a Gaussian output of central wavelength 1030 nm, average power 10 W, pulse duration ∼250 fs, and pulse repetition rate 50 kHz. For a typical experiment involving Bessel beams, the linearly polarized Gaussian beam was transformed into a non-diffracting Bessel beam by passing through an axicon mounted 5 cm before a focusing plano–convex lens of 5 cm focal length. The non-diffracting Bessel beam generated by the axicon (having a large numerical aperture, generating small Bessel zones of 30 μm period) is more tightly focusable, and lines that are 17 times thinner and ∼4 times deeper than those produced by the Gaussian beam, could be inscribed under identical scan conditions (ESI Note 1†). The samples were mounted on a dual-axis, motorized linear XY-translation stage (Thorlabs, DRV250 – 50 mm stepper motor drive) with control software to ensure precise positioning and processing of the sample surface. The scan parameters were adjusted for each sample to achieve the desired morphology without significantly compromising the optical absorbance. Parallel microchannels comprising hierarchical structures were inscribed by multiple laser scans. Each line on Mo was scanned 4 times at 3 mm s^−1^, and, on Al, 6 times at 10 mm s^−1^ before moving to the next adjacent line. The line spacing was maintained at 60 μm and 40 μm for the Gaussian and Bessel–Gauss beams, respectively.

### 2.2. Characterization

The surface morphology and cross-sectional analysis were performed with an FE-SEM (TESCAN-MAGNA, Czech Republic). Elemental composition was confirmed with an EDS detector (ULTIM MAX, Oxford Instruments, UK) installed with a FEM-SEM. IR measurements were performed with a FTIR spectrophotometer (IRTracer-100 fitted with MIRacle 10 Single reflection ATR, Shimadzu, Japan). Further chemical and structural analyses as well as investigation of the hydrogen bonding states were performed with Raman spectroscopy. Raman analysis was performed with a confocal Raman imaging microscope (alpha300 R, WITec, Germany) employing a 532× nm green laser and a 10× objective lens. The spectral range of analysis was limited to 160–1100 cm^−1^. The Raman spectra for all the samples were acquired with identical laser power and the same acquisition time. Further chemical analysis of the top surface (∼10 nm) was carried out with X-ray photoelectron spectroscopy (Nexsa G2, Thermo Scientific) using Al Kα X-rays (*hv* = 1486.6 eV). The energy resolution for the survey and high resolution spectra was 1 and 0.1 eV, respectively. The survey and high resolution plots represent average counts per s recorded over 3 and 5 scans, respectively. Depth profiling was performed by recording XPS spectra over multiple depth levels sputter-etched with Ar^+^ ions from a monoatomic etcher (MAGCIS) operated at 4 keV for 30 s. The curve fitting and linear background subtraction were performed with OriginPro 2021b (version: 9.8.5.204 (Academic)). Optical reflectance/absorbance was measured in the 250–2000 nm spectral range using a UV-vis-NIR spectrophotometer (UV-3600i Plus, Shimadzu, Japan). Total reflectance was measured with an integrating sphere relative to a highly diffuse reflecting BaSO_4_ reference, and the absorbance was calculated from the total reflectance values. A metallurgical microscope (EQ-MM500T-USB, MTI) with a CMOS camera was used to elucidate the observable changes in the surface appearance upon wicking with 3.5 wt% (aq) NaCl solution. Microscopy was conducted in reflectance mode with a 20× objective lens. Thermal images were captured using a thermal imaging camera (FLIR, E8 Pro, USA). Water hydrophilicity and contact angle measurements were performed with a drop shape analyzer (DSA100M, Kruss GmbH). Electrochemical measurements were performed with a potentiostat/galvanostat (VIONIC, Metrohm, Switzerland). The samples were tested in both DI water and 3.5 wt% NaCl solution with Ag/AgCl (3 M) and Pt as the reference and counter electrodes, respectively.

### 2.3. Interfacial solar evaporation experiments

**Fig. 2:**
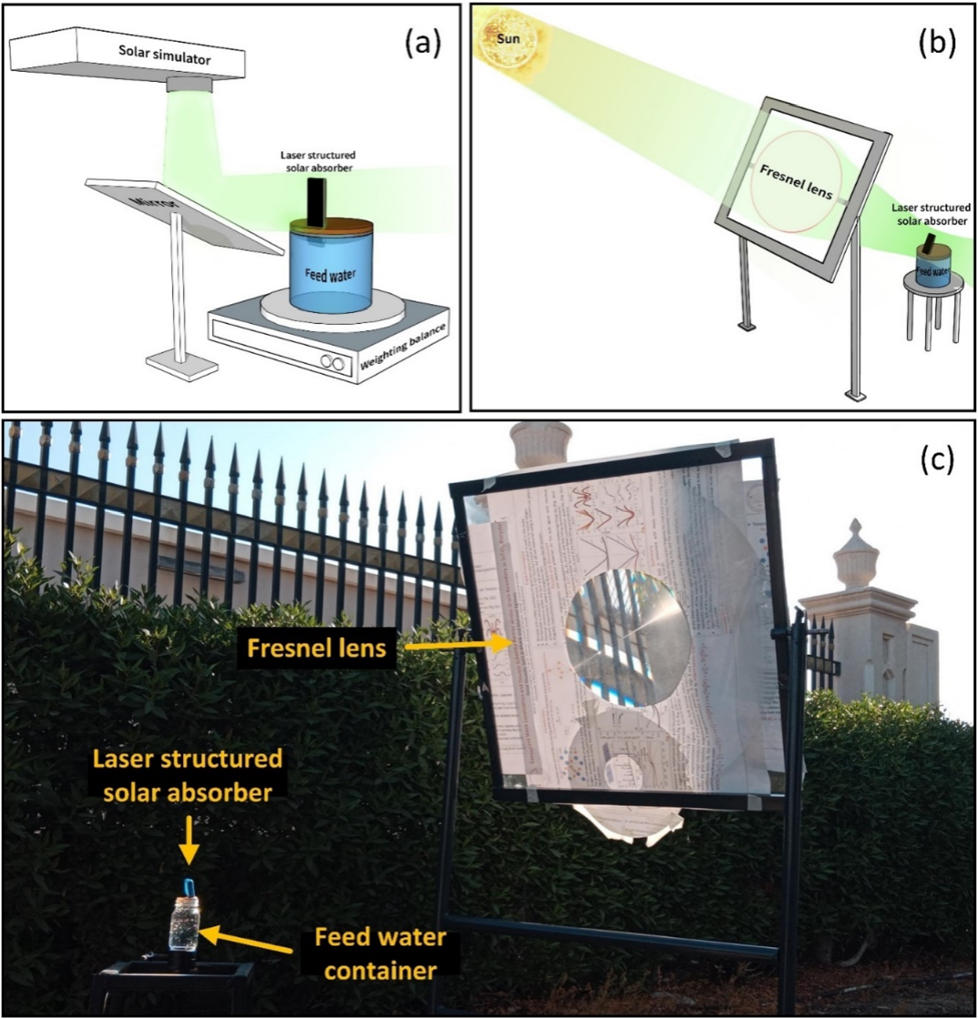
Schematic illustration of interfacial evaporation using a (a) solar simulator and (b) outdoor experiments under concentrated solar irradiation. (c) A photograph of the schematic illustration in (b).

A solar simulator (SL-50A-WS, Sciencetech, Canada) was used to perform evaporation experiments under 1 sun irradiance (1000 W m^−2^) with an AM 1.5G solar spectrum. The beam was extended horizontally using a high-reflectance mirror (Fig. 2(a)). The simulated light was calibrated at the test position to ensure 1 sun irradiance using an NREL-certified reference solar cell and a source measure unit (2450, Keithley, USA). The samples were mounted perpendicular to the beam direction on a cork holder with ∼3 mm of the samples submersed into the feed water. The samples were insulated from all sides (excluding the exposed absorber side) using Styrofoam to reduce convective heat losses to the surroundings. A laboratory weighing balance was used to quantify the mass loss from the water reservoir as a function of time.

The 2 and 3 sun experiments were performed outdoors under the sun at the American University of Sharjah, University City, Sharjah, United Arab Emirates (25°18′28.7′′N, 55°29′27.5′′E). Solar light was concentrated using a Fresnel lens to achieve the desired irradiance measured with a power meter (Ophir Optronics Solutions, Nova II) and irradiated onto the absorber that was open to the atmosphere. To demonstrate solar tracking, the evaporator inclination angle was adjusted according to the solar zenith angle, ensuring that light fell perpendicularly on the evaporator at various points of time throughout the day. Each measurement was performed over 5 minutes, and the sample orientation was readjusted to match the solar zenith angle before the next measurement. Multiple readings were taken throughout the day, and the average values are reported here. During these experiments, the maximum ambient temperature was 42 °C, whereas the average humidity and wind speed were 30% and 14 km h^−1^, respectively. The schematic diagram and a photograph of the outdoors experimental setup are shown in Fig. 2(b) and (c), respectively.

## 3. Results and discussion

### 3.1. Morphological analysis

**Fig. 3:**
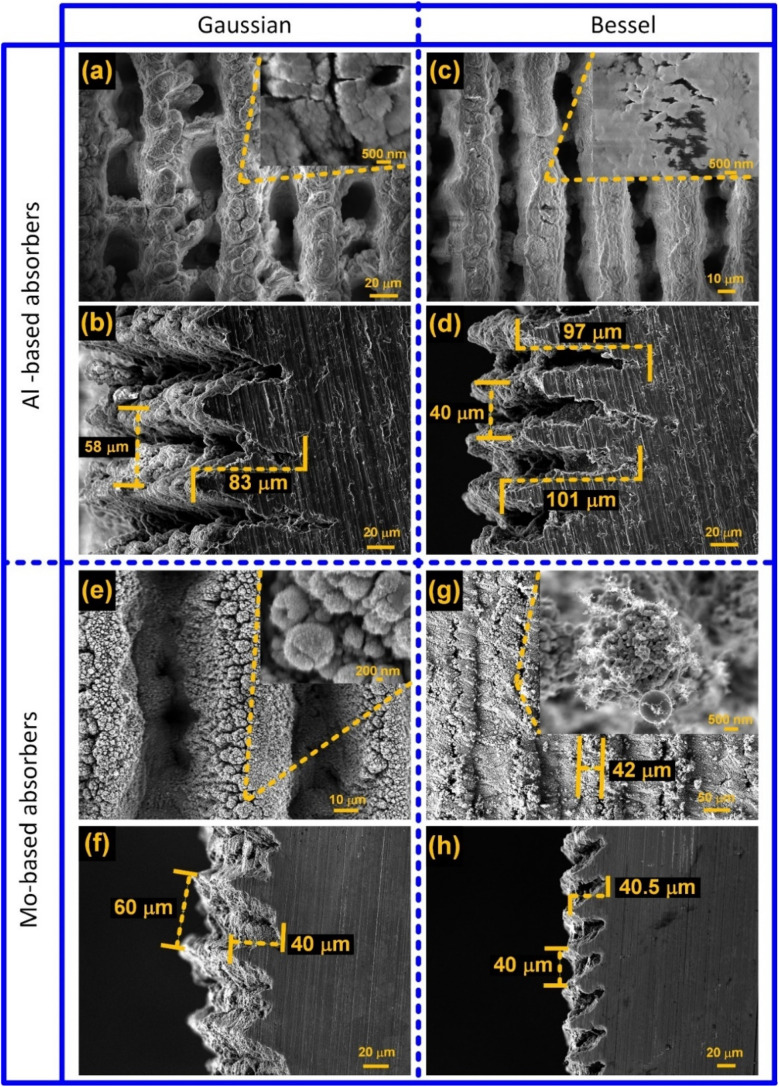
(a–d) SEM micrographs of Al samples structured with femtosecond Gaussian and Bessel beams: (a and c) top views with zoomed-in insets, and (b and d) the corresponding cross-sectional views. (e–h) SEM micrographs of Mo structured with femtosecond Gaussian and Bessel beams: (e and g) top views with zoomed-in insets, and (f and h) the corresponding cross-sectional views.

Four different solar evaporators were prepared: (1) Al-structured using a Gaussian beam, (2) Al- structured using a Bessel–Gauss beam, (3) Mo-structured using a Gaussian beam, and (4) Mo-structured using a Bessel–Gauss beam. The SEM micrographs of these solar evaporators are shown in Fig. 3. As evident from the SEM images, multiple parallel microchannels comprising hierarchical structures were inscribed on the surface of these evaporators with either Gaussian or Bessel–Guass beams. These open microchannels are crucial for achieving strong capillary action, enabling the rapid vertical supply of water without the need for active pumping.^54^ The microchannels, due to their open architecture, also allow for quick redeployment of the solar evaporators after salt accumulation by quickly rinsing off the accumulated salts with water. A comparison of the microchannels in Fig. 3(a and c) reveals that, unlike the Al-Gaussian evaporator, the microchannels in the Al-Bessel solar evaporator were more continuous, deeper, and thinner. This is because the Bessel–Gauss beam concentrates most of its energy at the center and can be focused more tightly than the Gaussian beam (ESI Note 1†). The cross-sectional micrographs in Fig. 3(b and d) show that the Bessel–Gauss beam can drill deeper, narrower, and more densely packed microchannels than the Gaussian beam. Fig. 3(a and c) and their zoomed-in insets also depict the presence of randomly distributed micro- and nanoparticles on the surface. The hierarchical structuring can be viewed as nanofeatures superimposed on microfeatures. The nanoparticles and the hierarchical morphology help improve solar energy harvesting. The randomly distributed nanoparticles on the surface of the solar evaporators enable solar light absorption *via* excitation of localized surface plasmon resonance.^11,55,56^ In addition, the laser-induced structural features trap solar light through multiple reflections,^11,57^ imparting high solar absorbance to both Al-Gaussian and Al-Bessel–Gauss solar evaporators.

The SEM images of Mo-Gaussian and Mo-Bessel–Gauss are shown in Fig. 3(e and g). Compared to Al solar evaporators, Mo solar evaporators showed a higher content of nanoparticles and a reduced degree of sintering effect, irrespective of the laser beam used. This is because Mo is an intrinsically high melting point material (2623 °C) and could not coalesce as easily as Al (melting point: 660.3 °C) during laser structuring. As in the case of Al, the microchannels in the Mo-Bessel–Gauss samples are thinner compared to those in the Mo-Gaussian sample due to differences in the focal spot size and distinct intensity profiles of the Gaussian and Bessel beams (Fig. 1). The Bessel beam concentrates most of its energy tightly at the center, causing more localized ablation. A footprint of this energy localization can be observed in the Mo-Bessel sample (Fig. 3(g and h)), with the ablation debris accumulated in the microchannels, thus constituting networks of nanochannels atop the microchannels. This effect was selectively evident in the Mo-Bessel sample because the tightly focused beam does not provide sufficient energy to significantly reablate the ablation debris (from previous scans), which settle in the vicinity of a scanned line. This morphology is important because it does not significantly compromise the optical absorption or superwicking properties. Instead, it provides nanochannels for enhanced interfacial evaporation while simultaneously mitigating heat losses to bulk water by filling the microchannels with nanochannel networks. Such filled microchannels could not be observed in the Al-Bessel sample (Fig. 3(c and d)) because Al has a much lower melting point compared to Mo, making it easily ablatable at lower fluences. The same rationale applies to both Al-Gaussian (Fig. 3(a and b)) and Mo-Gaussian samples (Fig. 3(e and f)), where the Gaussian beam due to its wider energy distribution significantly reablates the neighboring ablation debris.

### 3.2. Surface chemistry and structural analyses

**Fig. 4:**
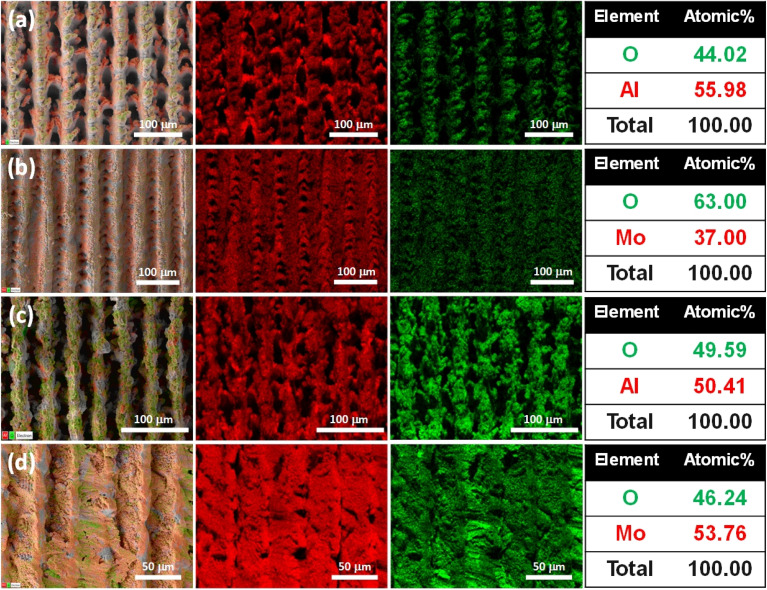
(From left to right) SEM layered micrograph, elemental mapping of the metal, *i.e.*, Al or Mo in red and O in green, and at% composition of femtosecond laser structured (a) Al-Gaussian, (b) Mo-Gaussian, (c) Al-Bessel–Gauss, and (d) Mo-Bessel Gauss solar evaporators.

Fig. 4(a–d) provide information about the elemental composition from EDS analysis of the structured Al and Mo solar evaporators. A considerable O content accounting for 44 at% can be observed spatially distributed across the Al-Gaussian surface (Fig. 4(a)). This significant O content is due to the ablation process in air, facilitating the formation of Al oxides. The Al to O at% ratio was confirmed to be 1 : 0.8; therefore, the O content is substoichiometric to that of Al_2_O_3_ (Al : O = 1 : 1.5). The ratio 1 : 0.8 equals 5 : 4 in whole numbers, and since there is no known stable configuration of Al_5_O_4_, the structured Al can be considered to primarily comprise substoichiometric Al oxides (AlO_*x*_).^58^ Similarly, the Al to O at% ratio for the Al-Bessel–Gauss sample was ∼1 : 1 (Fig. 4(c)), indicating substoichiometric AlO_*x*_. The elemental composition of Mo-Gaussian (Fig. 4(b)) shows that O is uniformly distributed along the structured surface and constitutes around 63 at% of the total composition. The Mo to O atomic ratio equals 1 : 1.7, which is significantly lower than that of stoichiometric MoO_3_. It is, therefore, believed to comprise several substoichiometric Mo oxides (MoO_*x*_).^59,60^ The same argument applies to the Mo-Bessel–Gauss sample (Fig. 4(d)) exhibiting substoichiometric O content. XPS survey spectra (ESI Note 4†) also confirm the same chemical constituents occupying the surface.

Surface chemistry imparts superwicking characteristics to these solar evaporators (refer to ESI Video 1† for freshly prepared AlO_*x*_ and ESI Video 2† for 50 days old MoO_*x*_ solar evaporators). The presence of considerable O amount on the surface makes the solar evaporators hydrophilic, whereas the hierarchical structures augment the hydrophilicity by rendering the surface superhydrophilic. The superhydrophilic micro-/nanocapillaries, therefore, impart superwicking properties to the evaporators. It was also observed that all the solar evaporators exhibited superwicking behavior irrespective of their orientation, implying compatibility with solar tracking and concentrated solar exposure throughout the day.

**Fig. 5:**
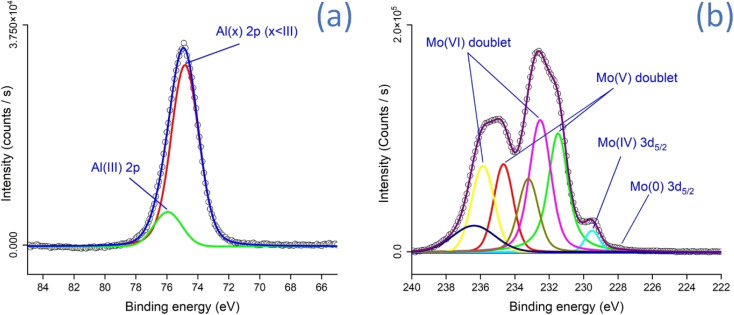
X-ray photoelectron spectroscopy of (a) AlO_*x*_ and (b) MoO_*x*_.

IR spectroscopy (ESI Note 5†) of Al-Gaussian and Al-Bessel samples reveals the presence of amorphous aluminum oxides and hydroxides in these samples. Similarly, the IR spectra of Mo-Gaussian and Mo-Bessel samples reveal the existence of mixed Mo oxide phases in these samples. Further insights into the structure and chemical composition of the surface were acquired with Raman analysis (ESI Note 6†). The surface of both Al-Gaussian and Al-Bessel-Gauss samples is primarily composed of amorphous Al oxides (AlO_*x*_). Similarly, substoichiometric Mo oxides (MoO_*x*_, *x* < 3) are found to dominate the surface of both Mo-Gaussian and Mo-Bessel samples. These findings about the surface chemical composition were further confirmed with high resolution XPS (Fig. 5) analysis. The peaks at 74.8 eV and 75.9 eV (Fig. 5(a)) correspond to the different oxidation states of Al. The peak at 74.8 eV corresponds to a mixed oxidation state, AlO_*x*_, with an oxidation state less than that of stoichiometric Al_2_O_3_.^61^ AlO_*x*_, which we previously introduced as amorphous Al oxide constitutes the major portion on the surface. Furthermore, the relatively smaller peak at 75.9 eV is assigned to Al(iii) in Al_2_O_3_.^61,62^ Depth profiling (ESI Note 7†) reveals that the AlO_*x*_ component increases as we move down the surface, which is anticipated due to the reduced exposure of the sub-surface to oxygen during and after the laser treatment. The above findings indicate that amorphous AlO_*x*_ is the major constituent of our Al-based evaporators. Similarly, in the case of Mo-based evaporators, several XPS peaks corresponding to mixed oxidation states were identified (Fig. 5(b)). A minor fraction of metallic Mo was also detected, as confirmed by the presence of the Mo(0) 3d_5_/_2_ peak at 227.9 eV. Substoichiometric Mo(iv) 3d_5/2_ could be located at 229.3 eV. The more dominant 3d_5/2_ and 3d_3/2_ doublets of Mo(v) and Mo(vi) were identified at 231.5 eV & 234.6 eV, and 232.5 eV & 235.8 eV, respectively.^63–65^ Below the top surface, the relative content of lower oxidation states, such as Mo(0) and Mo(iv), increases with depth (ESI Note 7†). This is attributed to the reduced exposure of the underlying material to oxygen during and after the laser treatment.

Mo(iv), present as MoO_2_, is a highly substoichiometric form of MoO_*x*_ (*x* < 3) that features delocalized d-orbital electrons. It has Mo–Mo metallic bonds and exhibits semimetallic characteristics.^66^ Moreover, the Fermi level in MoO_2_ occupies the same band as the d-orbitals.^67^ These delocalized d-orbital electrons are expected to influence the adsorption of water and salt ions on hydrophilic MoO_*x*_ in the desalination experiments.^68,69^ Additionally, the d-orbital electrons are responsible for the surface plasmon resonance effect, which enhances the optical absorption of MoO_2_ in the visible to NIR region.^44^

### 3.3. Optical response

Besides exhibiting superwicking properties, broadband optical absorbance was also observed for the fabricated black absorbers. UV-vis-NIR spectrophotometry was performed to analyze the optical absorption behavior of the solar absorbers within the spectral range of AM 1.5G. Both pristine Al and Mo surfaces exhibited very high total reflectance. The total reflectance measured for metallic Al and Mo exceeds that of the BaSO_4_ reference (Fig. S12†).

**Fig. 6:**
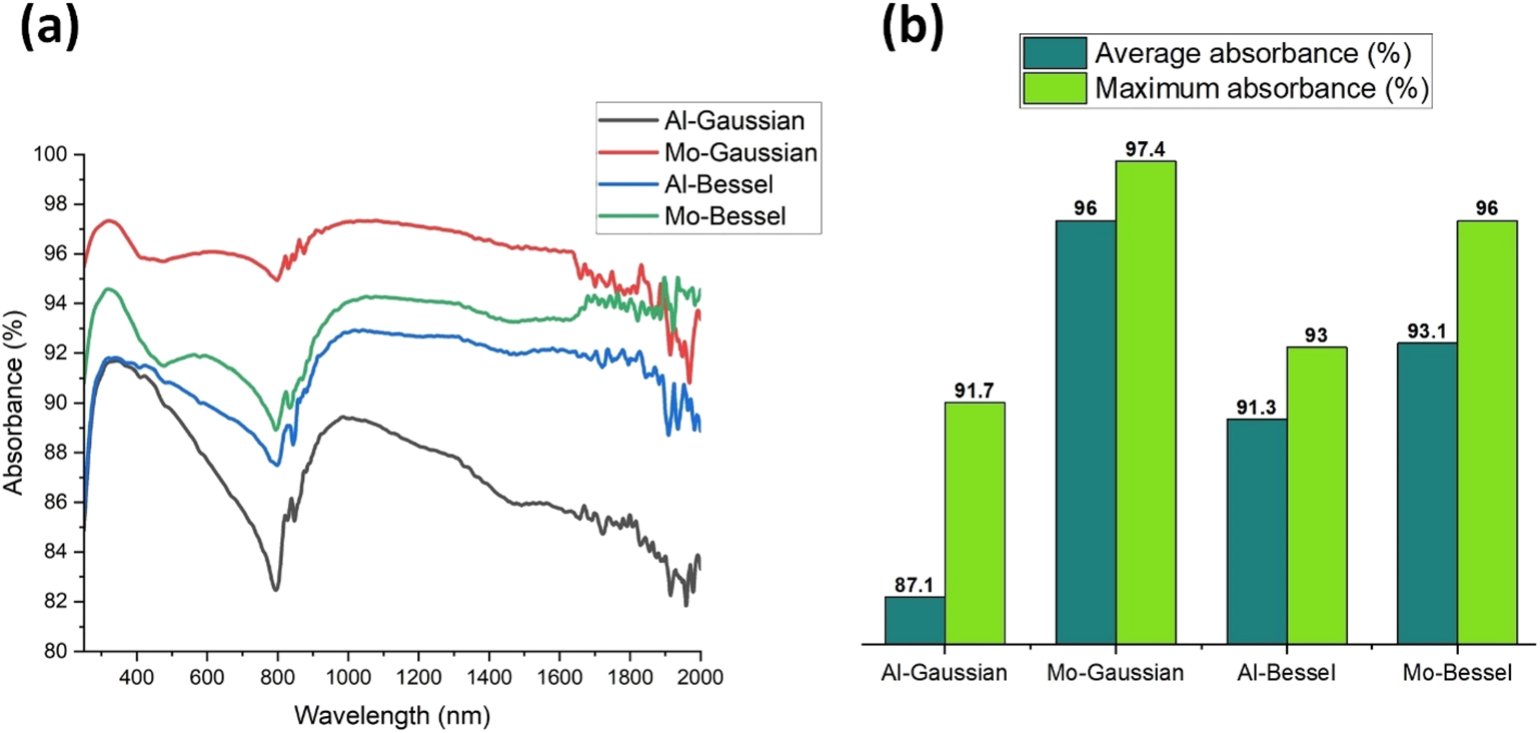
(a) UV-vis-NIR spectrophotometry % absorbance data of Al and Mo structured with femtosecond Gaussian and Bessel–Gauss beams, and (b) the corresponding average and maximum % absorbance for the structured solar absorbers recorded in the 250–2000 nm spectral range.

The absorbance recorded for the laser-structured solar absorbers is shown in Fig. 6(a). All the samples depicted considerable broadband absorbance over the spectral range from 250–2000 nm. Fig. 6(b) provides the numerical interpretation of Fig. 6(a) in terms of the average and maximum absorbance recorded. Al-Gaussian depicted the least average absorbance of 87.1%, followed by Al-Bessel with 91.3% average absorbance. On the other hand, Mo-Gaussian depicted the highest average absorbance of 96%, followed by Mo-Bessel with 93.1% average absorbance.

The significant enhancement in optical absorbance arises due to the highly rough surface morphology achieved with laser treatment. The microgrooves decorated with hierarchical structures (Fig. 3) are responsible for causing high diffuse reflectance, multiple reflections, and subsequent absorption of a large fraction of the incident rays by the absorber. The reason for the improved absorbance of Al-Bessel relative to Al-Gaussian is the denser, thinner, and deeper grooves inscribed by the Bessel beam. On the other hand, the MoO_*x*_ samples exhibit higher absorption than AlO_*x*_ primarily due to the availability of a narrow and dense d-band overlapping with the s–p conduction band in MoO_*x*_.^70^

### 3.4. Interfacial solar desalination performance

**Fig. 7:**
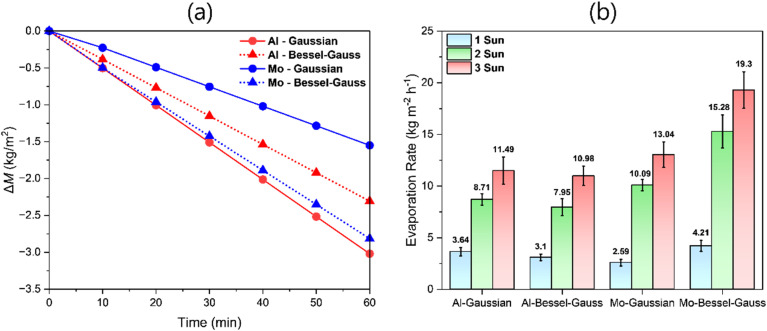
Interfacial solar evaporation of the prepared solar evaporators. (a) The measured change in the mass of DI water over time under simulated solar irradiance of 1 sun. (b) The average evaporation rate of saline water at different solar concentrations under real field (outdoor) conditions.

Fig. 7(a) compares the area normalized change in DI water mass *vs.* time for the different solar absorbers under 1 sun irradiance in a controlled laboratory environment. In each case, the mass of DI water declined linearly with time, implying a constant evaporation rate over the measurement period. Our results indicated that with DI water feed, Al-Gaussian produced the highest evaporation rate (3.02 kg m^−2^ h^−1^) despite exhibiting the lowest solar absorbance among the four samples (Fig. 7). The evaporation rate of Al-Bessel (2.31 kg m^−2^ h^−1^) was lower than that of Al-Gaussian, although Al-Bessel exhibited higher absorbance than Al-Gaussian. In contrast, Mo-Gaussian, which exhibits the highest optical absorbance, has the lowest evaporation rate (1.55 kg m^−2^ h^−1^). Similarly, Mo-Bessel absorbs more than Al-Gaussian; however, the evaporation rate on Mo-Bessel (2.81 kg m^−2^ h^−1^) was lower than that on Al-Gaussian (3.02 kg m^−2^ h^−1^). These observations can be explained by disentangling the individual roles of morphology and optical absorbance in interfacial solar evaporation. While high optical absorbance is essential, the pronounced effect of surface morphology and interactions between the solar evaporators and water at the evaporator/water interface can play a crucial role in dictating the evaporation rates.

When considering the surface morphology consisting of microchannels, it can be said that MoO_*x*_ holds DI water more strongly at the evaporator/water interface than AlO_*x*_ does. This is attributed to the much stronger adsorption of water on MoO_*x*_ than on AlO_*x*_,^71,72^ which has important implications for interfacial evaporation where the morphology of the water channels is more critical than the optical absorbance for evaporators attaining ∼87% or above absorbance under 1 sun illumination. Channels with smaller free volumes hold fewer water bodies and are more efficient evaporators than channels with larger free volumes. Observations supporting this claim are the higher evaporation rates observed for Al-Gaussian (with shallower channels, partially filled with nanoparticles) compared to Al-Bessel (with deeper and unfilled channels) (Fig. 3(a–d)). Also, the unique morphology of Mo-Bessel consisting of nanochannels superimposing microchannels resulted in a high evaporation rate compared to Mo-Gaussian with wider and unfilled microchannels (Fig. 3(e–h)). This again highlights the importance of morphology and the interactions at the evaporator/water interface in dictating the evaporation rate. These interactions are further elucidated in later sections.

When experiments were performed with saline water under controlled laboratory conditions under 1 sun irradiance, the evaporation rates on Al-Gaussian, Mo-Gaussian, Al-Bessel, and Mo-Bessel solar evaporators were 1.89, 1.23, 1.79, and 1.92 kg m^−2^ h^−1^, respectively. This indicated that, regardless of the evaporator, the evaporation rate of saline water is always lower than that of DI water at 1 sun. This behavior was anticipated and can be associated with boiling point elevation and interface modification due to salinity, which is discussed in detail in the section on the electrochemical analysis of the interfaces. Moreover, MoO_*x*_ evaporators can attain higher evaporation rates, partly due to the higher photothermal temperatures (ESI Note 9†) attained due to their relatively higher optical absorption (Fig. 6). Fig. 7(b) compares the average evaporation rates achieved with 3.5 wt% saline water on the MoO_*x*_ and AlO_*x*_ evaporators under real field conditions at 1, 2, and 3 suns. Fig. 7(b) also indicates that regardless of the evaporator, the evaporation rate changes non-linearly with a linear increase in solar irradiance from 1 to 3 suns. The change in the evaporation rate is more pronounced when solar irradiance increases from 1 to 2 suns, while it is less significant between 2 and 3 suns. Moreover, Mo-Bessel depicts the highest evaporation rate with saline water at 1, 2, and 3 suns. Mo-Gaussian surpasses both Al-Gaussian and Al-Bessel evaporators at 2 and 3 suns. It is evident that both salinity and solar concentration selectively favor evaporation on MoO_*x*_. The origin of the rapid and non-linear increase in the total evaporation rate on MoO_*x*_ is the stronger plasmonic effect of delocalized electrons in MoO_*x*_ under concentrated solar irradiation, activating stronger interfacial evaporation. This has been discussed in more detail in the section on the electrochemical analysis of the interfaces. In contrast, the less rapid non-linear increment from 2 to 3 suns is attributed to excessive salt accumulation hindering efficient optical absorption and amplifying optical reflection from the accumulated NaCl crystals. Efficient evaporators such as Mo-Bessel require regular surface cleaning to remove accumulated salt. Luckily, rinsing with water easily washes off most of the accumulated salt from the evaporator.

In outdoor experiments, multiple factors influence the evaporation rate, especially the wind speed, ambient temperature, and humidity. Under controlled laboratory conditions at 1 sun irradiance, the evaporation rates on Al-Gaussian, Mo-Gaussian, Al-Bessel, and Mo-Bessel solar evaporators were 1.89, 1.23, 1.79, and 1.92 kg m^−2^ h^−1^, respectively. Comparing these results with the outdoor results (Fig. 7(b)), around ∼42–54% of the evaporation rate is attributable to wind and ambient conditions under 1 sun. It is therefore imperative that approximately half of the total evaporation rates recorded in Fig. 7(b) under 1, 2, and 3 suns are attributable to wind and higher outdoor temperature. Since wind speed fluctuates continuously, the evaporation rate varies accordingly.

### 3.5. Latent heat of vaporization and energy conversion efficiency

**Fig. 8:**
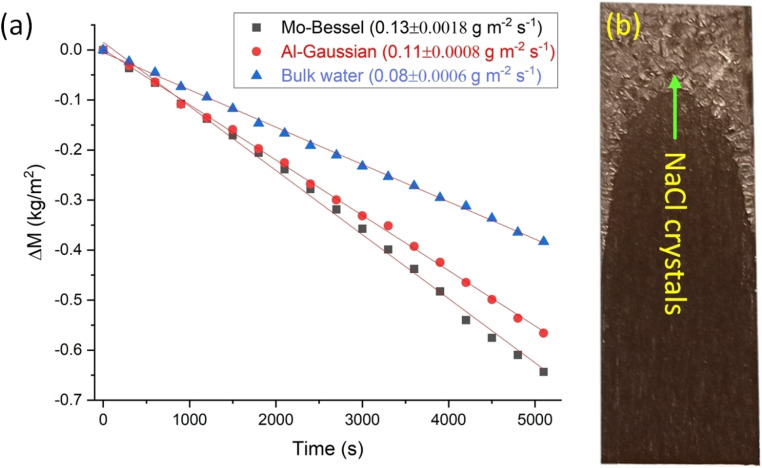
(a) Water evaporation in the dark normalized to geometric surface area *vs.* time. Linear fitting was applied to extract the slope which is the geometric area normalized evaporation rate (*ṁ*_geo_). (b) A camera captured image of MoO_*x*_ (*i.e.*, Mo-Bessel) after 1.4 hours of evaporation in the dark.

Fig. 8(a) depicts plots of saline water evaporation normalized to geometric surface area *vs.* time observed for AlO_*x*_, MoO_*x*_, and bulk water at 20 °C under dark conditions. Applying linear fitting, the geometric area normalized water evaporation rates (*ṁ*_geo_) measured for bulk water, AlO_*x*_, and MoO_*x*_ are 0.08 ± 0.0006 g m^−2^ s^−1^, 0.11 ± 0.0008 g m^−2^ s^−1^, and 0.13 ± 0.0018 g m^−2^ s^−1^, respectively. The respective real surface areas of water on AlO_*x*_ and MoO_*x*_ are 82.5% and 85% of that of the bulk water with equal geometric surface area, respectively (ESI Note 10†). Therefore, the real area normalized saline water evaporation rates (*ṁ*) on AlO_*x*_ and MoO_*x*_ are 0.133 ± 0.001 g m^−2^ s^−1^, and 0.153 ± 0.002 g m^−2^ s^−1^, respectively.

The enthalpy of vaporization (*H*_LV_) of saline water over AlO_*x*_ and MoO_*x*_ can be computed by assuming that bulk water is an ideal device operating at 100% energy conversion efficiency in the dark. If the respective dark evaporation rates (*ṁ*) of the ideal bulk solution device (*ṁ*_bulk_) and the evaporators (*i.e.*, *ṁ*_AlO_*x*__ and *ṁ*_MoO_*x*__) are known, then the latent heat of vaporization of saline water over AlO_*x*_
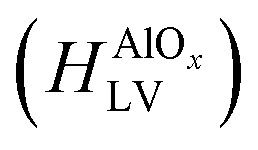
 and MoO_*x*_
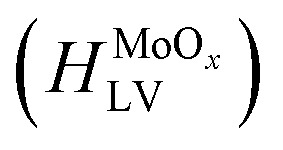
 can be computed using the following relation.^73^
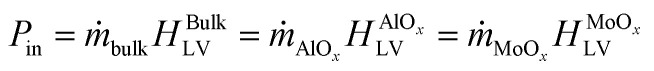
Here, *P*_in_ is the uniform power per unit area available to the ideal bulk solution and the evaporators under dark conditions. *H*^bulk^_LV_ of 3.5 wt% NaCl (aq) solution at 20 °C and 0.1 MPa has been reported to be *ca.* 2360 kJ kg^−1^.^74^ From the above data, 
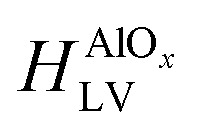
 and 
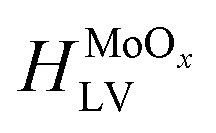
 were computed to be 1420 kJ kg^−1^ and 1234 kJ kg^−1^, respectively. These amount to *ca.* 40% and 48% reduction in the vaporization enthalpy of saline water over the AlO_*x*_ and MoO_*x*_ evaporators, respectively. This significant reduction in the vaporization enthalpy is the basic reason for the enhanced evaporation rates observed in this study.

The energy conversion efficiency (η) of a typical solar evaporator (under controlled laboratory conditions) may be computed using the following expression.^53,73,75^
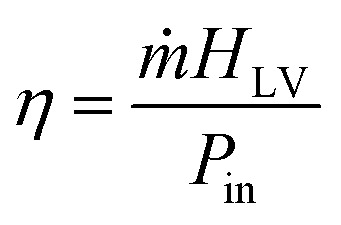

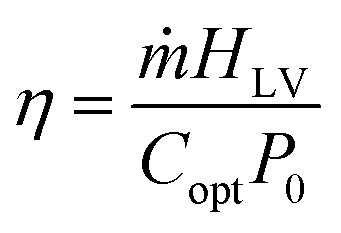
where, *P*_in_ is the solar irradiance (illumination intensity) and is given by *C*_opt_*P*_0_,where, *C*_opt_ is the optical concentration (with a numerical value of 1, 2, and 3 for 1, 2, and 3 suns, respectively), and *P*_0_ is 1000 W m^−2^. The above formula does not account for environmental factors and cannot be applied directly to the values from Fig. 7(b) to compute the solar conversion efficiency. As discussed above, the solar energy alone (under controlled lab conditions) constitutes around 50% (∼46–52%) of the total outdoor evaporation rate and the remaining is due to environmental factors (temperature 42 °C, humidity 30%, and wind speed 14 km h^−1^). By negating the ambient effects, we compute the solar efficiency of AlO_*x*_ to be around 75%, 89%, and 78% at 1, 2, and 3 suns, respectively. Whereas, for MoO_*x*_, the efficiency comes out to be 66%, 119%, 101% at 1, 2, and 3 suns, respectively. Efficiency reduction at 3 suns compared to 2 suns has been discussed previously and has been associated with excessive salt accumulation. Whereas, efficiency above 100% indicates that at higher evaporation rates under concentrated solar irradiation, the contribution of ambient effects is even larger than 50% and shall be accounted for in outdoor experiments involving higher evaporation rates.

### 3.6. Raman spectroscopy of hydrogen bonds

The observed differences in the evaporation rates of DI water can be explained by the interaction of water with the solar evaporator surface, which in turn, can affect the local structure of water molecules and the hydrogen bonding state.^76^ To assess the local structure of water molecules close to the evaporator interface, Raman spectroscopy was performed for bulk DI water and water within the solar evaporators' capillaries (ESI Note 11†). The OH stretching vibration in Raman spectra for bulk DI water and water at the interface of the solar evaporators were deconvoluted into five Gaussian sub-peaks, and the relative proportions of different states of hydrogen bonding were calculated based on the sub-peak integral area^77^ (presented in Table S4†).

In general, for a water molecule, the hydrogen bonding network formed can be characterized by its interactions with the neighboring molecules acting either as a proton donor (D), a proton acceptor (A), or both.^77^ This way five types of hydrogen bonded motifs have been reported, namely, donor acceptor–acceptor (DAA-OH), donor–donor acceptor (DDA-OH), donor acceptor (DA-OH) donor–donor acceptor–acceptor (DDAA-OH), and free water molecules (free-OH).^76,77^ In the OH stretching vibration region of the Raman spectrum, these appear as five sub-bands centered around 3005, 3226, 3434, 3573, and 3640 cm^−1^, respectively.^11^ In our case, DA-OH (chain or ring) and DDAA-OH (tetrahedral) species showed the highest contributions (57.3% and 30.4%, respectively) in bulk water and represented the primary types of hydrogen bonding states that were present. This is consistent with the results from previous studies.^11,77–79^ For DI water confined in the channels of solar evaporator, we observed alterations in the hydrogen bonding networks, and there was a significant increase in the DDA-OH networks in the case of Al-Gaussian, Al-Bessel–Gauss, and Mo-Bessel–Gauss samples. For DI water in the channels of the solar evaporators, the percentage of DDA-OH hydrogen bonding networks was higher compared to bulk DI water (only 6.4%). Al-Gaussian and Mo-Bessel–Gauss showed comparable percentages of the DDA-OH hydrogen bonding networks (>30%) and produced similar evaporation rates with DI water. In the case of Al-Bessel–Gauss, the percentage of DAA-OH hydrogen bonding networks was less compared to Al-Gaussian and Mo-Bessel–Gauss. As a result, the evaporation rate was lower. Mo-Gaussian produced the lowest evaporation rate with DI water. This was consistent with the observation that, with Mo-Gaussian, DI water possessed the smallest percentage of DAA-OH networks. It is known that DDA-OH networks, which are clusters of four water molecules, can bind more water molecules and have the tendency to form large-sized water clusters. The formation of water clusters reduces the enthalpy of vaporization compared to the conventional latent heat. This is because in large-sized water clusters, only the hydrogen bonds formed between the clusters and the evaporator surface need to be broken. Consequently, large-sized water clusters tend to escape collectively at a lower energy per unit mass compared to monomeric water molecules that require all individual hydrogen bonds to be broken.^11,80^ In the case of Al-Bessel–Gauss, the population of water clusters was not as high, which implied higher enthalpy of vaporization for DI water on the Al-Bessel–Gauss surface and, consequently, a lower evaporation rate compared to Al-Gaussian and Mo-Bessel–Gauss. For Mo-Gaussian, DI water possessed the smallest percentage of DAA-OH networks and, therefore, the lowest population of water clusters. This resulted in Mo-Gaussian producing the lowest evaporation rate with DI water. With the established DI water evaporation trends for different evaporators, we can now use these data as a reference to further explore the evaporation behavior of saline water on these evaporators.

Photothermal heating, morphology of the evaporator channels, and the salt water/evaporator interface are important from the design perspective and have been discussed below.^81,82^ The superior performance of MoO_*x*_ evaporators with 3.5 wt% saline water can be attributed to the changes in the hydrogen bonding states on the solar evaporator surface in the presence of salt ions. The deconvoluted Raman spectra of the OH stretching vibrations in saline water (ESI Note 11†) suggest that the percentage of DDA-OH hydrogen bonding networks is much higher for water confined in MoO_*x*_ evaporators (>75%) compared to AlO_*x*_ evaporators (∼15%). This distinction in the hydrogen bonding between the MoO_*x*_ and AlO_*x*_ evaporators is attributed to the delocalized d-orbital electrons on MoO_*x*_ responsible for strongly adsorbing the salt ions. We discuss this fact in more detail in the next section on electrochemical probing of the evaporator/water interface.

**Fig. 9:**
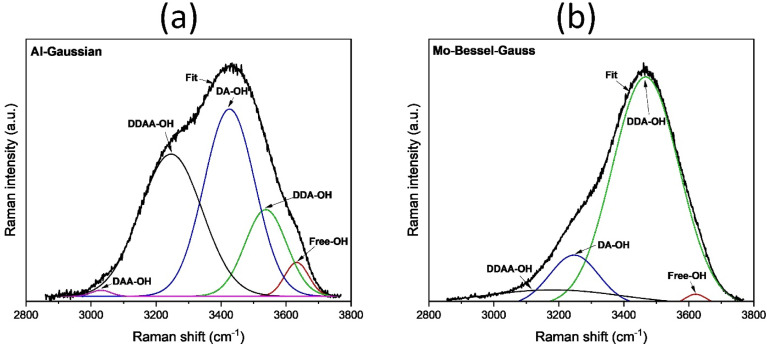
Deconvoluted OH stretching vibration in the Raman spectra for (a) Al-Gaussian and (b) Mo-Bessel–Gauss (with DAA-OH percentage zero) in 3.5 wt% NaCl solution.

Fig. 9 contrasts the deconvoluted Raman spectra of OH stretching for the best AlO_*x*_, *i.e.*, Al-Gaussian (DAA-OH percentage: 34%), and the best MoO_*x*_, *i.e.*, Mo-Bessel–Gauss (DAA-OH percentage: 76.3%) in saline water. These results imply a relative abundance of water clusters responsible for a reduction in the vaporization enthalpy, and a consequent increase in the evaporation rate for Mo-Bessel–Gauss in saline water. It is evident that besides the nanochannel superimposed microchannel morphology, the salinity is another factor favourably affecting the evaporation rate on MoO_*x*_ compared to AlO_*x*_. Due to the delocalized d-orbital electrons, MoO_*x*_ is anticipated to form an electrical double layer (EDL) different from that on AlO_*x*_ in saline water. The EDL is essentially an array of charged species, which comprise salt ions and water dipoles in our case, that align themselves along the (conducting) evaporator surface when immersed in saline water. The EDL typically comprises two layers of charged species (discussed in the next section), with one of the layers specifically adsorbing on the evaporator.^83^ The presence of the EDL is due to adsorption; therefore, it is imperative to investigate the EDL in saline water. In the following section, we electrochemically investigate the nature of the EDL that develops along the MoO_*x*_ and AlO_*x*_ evaporators.

### 3.7. Electrochemical analysis of the interfaces

So far, we have been able to confirm that morphology and salinity of water influence interfacial evaporation. Furthermore, it has been established that cluster evaporation, which leads to higher evaporation rates, is promoted in the presence of more interfaces and less bulk water. This is particularly evident for Mo-Bessel, where narrower nanochannels have replaced the broader Gaussian-induced microchannels. Raman studies of OH stretching vibrations supported this, indicating increased clustering and reduced free-molecular evaporation. This effect is more pronounced on MoO_*x*_ samples when tested in saline water. For example, with similar Gaussian morphologies of AlO_*x*_ and MoO_*x*_ in DI water, the AlO_*x*_ could vaporize water more easily than the MoO_*x*_ evaporator (Fig. 7(a)). However, the evaporation rates are almost equal in salt water at 1 sun (controlled lab conditions). At 2 and 3 suns, Mo-Gaussian performs better than Al-Gaussian. Due to the different morphology of Mo-Bessel, its evaporation rate marginally lags behind that of Al-Gaussian in DI water (Fig. 7(a)). It outperforms all samples in saline water (Fig. 7(b)). Below, we elaborate on why salt selectively improves the evaporation rate of MoO_*x*_ evaporators.

Salt addition elevates the boiling point of bulk water, such that 3.5 wt% NaCl has a boiling point elevation (BPE) of ∼0.33 °C relative to pure water at sea level at 25 °C (ESI Note 12†). The BPE is caused by additional interactions associated with the hydration of Na^+^ and Cl^−^ ions. The changes in the interfacial evaporation rate with salinity do not correlate linearly with the BPE of 0.33 °C (Fig. 7(a) and (b)). This is due to differences between bulk and interfacial evaporation mechanisms, where interfacial evaporation depends on the nature of the evaporator interface. Salt ions tend to adsorb strongly on the solid evaporators.^84^ A stable EDL appears due to a dynamic equilibrium of charge transfer between the evaporator and the adsorbed salt ions/water dipoles. Depending on the equilibrium position, net charges can accumulate on the electrode and in the solution phase, such that a potential appears across the EDL. Under equilibrium, no net current passes across the EDL, and therefore, this equilibrium potential is referred to as open circuit potential (OCP).^85^ The stronger the adsorption interaction, the easier the charge exchange between the evaporator and saline solution. Thus, a change in OCP can indicate changes in the EDL, corresponding to alterations in the specifically adsorbed species.

**Fig. 10:**
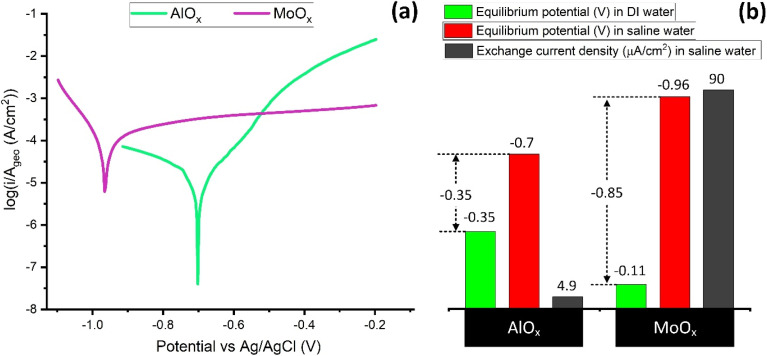
Comparison of the (a) Tafel plots in saline water and (b) the corresponding rest potentials (in saline as well as DI water) and exchange current densities of structured AlO_*x*_ and MoO_*x*_ samples.

The OCP of AlO_*x*_ and MoO_*x*_ in DI water (ESI Note 13†) and saline water (Fig. 10(a)) show interface modification with salt ion adsorption on the interface. For AlO_*x*_, the difference in OCP is −0.35 V, whereas for MoO_*x*_, the difference is −0.85 V (Fig. 10(b)). For MoO_*x*_, this difference in OCP is more than twice that of AlO_*x*_ and can be attributed to the difference in EDL emerging at the evaporator/solution interface (ESI Note 13†).

Here, it is important to consider the type and extent of adsorption interactions at the evaporator/electrolyte interface. In the case of AlO_*x*_ in DI water, there are two dominant modes of interaction between AlO_*x*_ and the electrolyte. One is the strong hydrogen bonding due to water dipoles interaction with AlO_*x*_. The second dominant interaction results from the partial negative oxygen (O(*δ*−)) of H_2_O interacting with the positive Al centers of AlO_*x*_. In the case of saline water, salt ions also compete for specific adsorption on the surface. The −0.35 V shift from −0.35 V in DI water to −0.7 V in saline water shows that salt ions have specifically adsorbed on AlO_*x*_. As suggested in the Bockris–Devanathan–Müller model, which is detailed in ref. 83 the adsorbed layer along the Helmholtz plane comprises adsorbed water dipoles and salt ions. This well-organized monolayer screens the secondary water and ions in the outer Gouy layers from directly interacting with the AlO_*x*_. Although this layer reduces the average direct contact between H_2_O and AlO_*x*_, it also introduces a concentration gradient with more salt ions near the AlO_*x*_ interface. This higher salt ion concentration is responsible for the BPE beyond ∼0.33 °C in the interfacial region and can be the primary factor contributing to the reduced evaporation rate of AlO_*x*_ in a saline environment.

In the case of MoO_*x*_, besides the interactions mentioned above, strong d-band interactions also exist between the positively charged metallic Mo centers and the adsorbates. Due to the d-band interactions, the adsorbates can adsorb more strongly on MoO_*x*_, resulting in higher heats of adsorption.^84^ This is the primary reason for the relatively lower evaporation rates observed on MoO_*x*_ in DI water. In saline water, the large OCP shift of −0.85 V and the higher exchange current density of 90 μA cm^−2^ indicate stronger salt ions adsorption on the interface. Due to the higher competitive specific adsorption of salt ions (as evidenced by the higher potential shift), the Helmholtz layer has a better screening effect and strongly limits the direct water contact with the MoO_*x*_. This releases more water molecules from direct d-band interaction with MoO_*x*_. In this case, the water favoring rapid evaporation occupies the Gouy layer.

Along with the screening effect at the interface, the excess local salt concentration promotes BPE to retard the evaporation rate as well. The interfacial evaporation rate thus represents a balance between d-band screening and BPEs. We observed that the evaporation rate of Mo-Gaussian was slightly lower than that of Al-Gaussian at 1 sun but surpassed Al-Gaussian at 2 and 3 suns. Mo-Bessel outperformed all the samples at 1, 2, and 3 suns in saline water. The origin of the significantly improved MoO_*x*_ performance at multiple suns is the provision of sufficient energy to overcome the d-band interactions of the remaining specifically adsorbed water along the Helmholtz plane. The favorably modified interface, the enhanced optical absorbance by the MoO_*x*_ delocalized d-band electrons due to a stronger plasmonic effect under concentrated solar irradiation, and preferred interface geometry (*e.g.*, Mo-Bessel), resulted in evaporators with the highest evaporation rates reported to date. Lastly, pertinent to corrosion resistance, the MoO_*x*_ type evaporators depict geometric corrosion current densities within an acceptable limit of 100 μA cm^−2^ (Fig. 10).^84^

Table 1 compares the performance of solar evaporators reported in this study with other interfacial evaporators reported elsewhere. The MoO_*x*_ solar evaporator produced with a femtosecond Bessel beam laser exhibited superior saline water evaporation rates, especially under concentrated solar. Under 1 sun (outdoor conditions), the observed evaporation rate was 4.21 kg m^−2^ h^−1^, exceeding most of the reported carbon, semiconductor, and plasmonic-based solar evaporators. Under 3 suns, the evaporation rate was remarkably high (19.3 kg m^−2^ h^−1^) compared to the carbonized wood reported by Liu *et al.*^86^ and the laser-treated Al reported by Singh *et al.*^11^ The present study, therefore, reports an efficient, physically stable, and easy-to-fabricate MoO_*x*_ solar evaporator that holds great promise for use in interfacial solar evaporation and offers new directions for future research in solar thermal water desalination.

**Table 1:** Comparison of different solar evaporators in interfacial solar evaporation

Evaporator material	Experimental conditions	Enthalpy of evaporation (J g^−1^)	Optical absorbance (%)	Evaporation rate (kg m^−2^ h^−1^)	Efficiency (%)	Reference
Carbon monoliths PET wastes	Intensity: 1 sun, feed: 3.5 wt% NaCl	—	92	0.99	63.5	24
Carbon sponge	Intensity: 1 sun, feed: 3.5 wt% NaCl	—	95–97	1.31	90.0	87
Multilayer carbon-fiber fabric	Intensity: 1 sun, feed: seawater	1094.28	97	3.39	96.69	88
Carbonized wood	Intensity: 3 suns, feed: 3.5 wt% NaCl	—	97	4.03	91.3	86
Carbon black	Intensity: 1 sun, feed: 3.5 wt% NaCl	—	96.5	2.31	—	89
SnSe on Ni foam	Intensity: 1 sun, feed: artificial seawater	—	89	0.85	—	90
MoO_*x*_ nanostructures	Intensity: 1 sun, feed: seawater	—	90	1.26	85.6	53
Au nanofilm	Intensity: 1 sun, feed: 150 g L^−1^ NaCl	—	80	0.88	49.5	91
Femtosecond laser-treated Al	Intensity: 3 suns, feed: pure water	1220	97	5.5	—	11
Femtosecond laser-treated Ti foam	Intensity: 1 sun, feed: pure water	—	97	1.79	90.0	41
Picosecond laser-treated Al	Intensity: 1 sun, feed: artificial seawater	541.25	85	1.24	67.0	42
Femtosecond laser (Gaussian beam)-treated Al	Intensity: 1 sun, feed: 3.5 wt% NaCl (aq)	1420	87.1	3.64	75	This work
Femtosecond laser (Gaussian beam)-treated Al	Intensity: 2 suns, feed: 3.5 wt% NaCl (aq)	—	87.1	8.71	—	This work
Femtosecond laser (Gaussian beam)-treated Al	Intensity: 3 suns, feed: 3.5 wt% NaCl (aq)	—	87.1	11.49	—	This work
Femtosecond laser (Bessel beam)-treated Mo	Intensity: 1 sun, feed: 3.5 wt% NaCl (aq)	1234	93.1	4.21	66	This work
Femtosecond laser (Bessel beam)-treated Mo	Intensity: 2 suns, feed: 3.5 wt% NaCl (aq)	—	93.1	15.28	—	This work
Femtosecond laser (Bessel beam)-treated Mo	Intensity: 3 suns, feed: 3.5 wt% NaCl (aq)	—	93.1	19.3	—	This work

## 4. Conclusion

Clean water is vital for sustaining life on Earth, and interfacial solar evaporation presents a promising approach for sustainable solar–thermal water desalination. However, its commercial viability is constrained by the lack of efficient and scalable solar thermal evaporators. To achieve high evaporation rates, evaporators must feature enhanced optical absorbance, superwicking properties, and effective interfacial water management. Current evaporators fall short of the evaporation rates needed for commercial success. Our study demonstrated the design of interfacial solar evaporators with enhanced evaporation rates, solar tracking capabilities, and compatibility with concentrated solar power. We demonstrated that meticulous structuring of metal surfaces to create metal oxide nanochannels overlaid on open microchannels results in some of the most efficient evaporators reported to date. We were able to produce Mo oxide nanochannels superimposed hierarchically on structured Mo microchannels (MoO_*x*_) using a tightly focused non-diffracting femtosecond laser Bessel beam. In contrast to the commonly-employed Gaussian beams, Bessel beams can be confined to tighter spots to produce relatively thinner channels with less ablation of the channel surroundings. Additionally, we conducted outdoor interfacial evaporation experiments with our evaporators under multiple suns to demonstrate their solar-tracking compatibility and effectiveness under concentrated solar irradiation. With the nanochannel-superimposed-microchannel morphology, we achieved average evaporation rates of saline water of approximately 19.3 kg m^2^ h^−1^ under 3 suns. This rate is nearly four times higher than the best values reported for previously studied solar evaporators. It has been shown that more than half of the total evaporation under concentrated solar irradiation may be due to ambient wind and high temperatures. The higher optical absorption of MoO_*x*_, which is also due to the plasmonic effect of delocalized d-orbital electrons, generates much higher thermal energies on MoO_*x*_. Such higher thermal energies provide the necessary activation to easily release the adsorbed water molecules from the strong d-orbital overlap. Additionally, the Mo d-band was found to play a significant role in modifying the Helmholtz layer on the MoO_*x*_ interface by specifically adsorbing a substantial amount of salt ions. These ions effectively shielded the secondary water in the Gouy layer from direct interactions with the d-band. The interface modification also had a pronounced effect on the hydrogen bonding near the interface, where water cluster evaporation was promoted by the salt ions that were strongly adsorbed on the fabricated nanochannels. Moreover, besides reporting the highest evaporation rates for any black metals to date, our work offers valuable guidelines for future research and development in the field of interfacial solar evaporation. With the rapid development in femtosecond laser technology over the past few years and their ease of use, these lasers can be utilized to efficiently produce large-area evaporator panels, making them highly suitable for large-scale interfacial solar evaporation.

## Author contributions

A. S. A. conceived and supervised the study. A. A. and M. Q. contributed equally to the work, conducted the experiments, and analyzed the data. A. A., M. Q., G. B., and P. A. P. set up the experimental apparatus. The final interpretations were carried out by A. A., M. Q., P. A. P., and A. S. A. All authors discussed the results and contributed to the final manuscript, which was drafted by A. A., M. Q., and A. S. A.

## Conflicts of interest

The authors declare no competing interests.

## Supplementary Material

NA-007-D5NA00249D-s001

NA-007-D5NA00249D-s002

NA-007-D5NA00249D-s003

## Data Availability

The data that support the findings of this study are available from the corresponding author upon reasonable request.
