# Supplementary material for: Enhanced interfacial solar desalination using nano-engineered MoOx photothermal evaporators
Source: Nanoscale Adv. 2025 May 19;7(14):4352–66. doi: 10.1039/d5na00249d (PMC12143300; doi:10.1039/d5na00249d)
Supplement: NA-007-D5NA00249D-s003 [file NA-007-D5NA00249D-s003.pdf]

Supplementary Information (SI) for Nanoscale Advances.
This journal is © The Royal Society of Chemistry 2025

Supporting Information

Enhanced Interfacial Solar Desalination Using Nano-Engineered MoO_x Photothermal Evaporator

Asghar Ali[1,2], Muhammad Qasim[1,3], Piotr A. Piatkowski[1,4] , Ganjaboy Boltaev[2,4] , Andra N. K. Reddy[2,4], and Ali S. Alnaser [1,2,4*]

[1]Materials Science and Engineering Program, College of Arts and Sciences, American University of Sharjah, Sharjah 26666, United Arab Emirates

[2]Department of Physics, American University of Sharjah, Sharjah 26666, United Arab Emirates

[3]Department of Chemical and Biological Engineering, American University of Sharjah, Sharjah 26666, United Arab Emirates

[4]Materials Research Center, American University of Sharjah, Sharjah 26666, United Arab Emirates

[*] Corresponding author

E-mail address: aalnaser@aus.edu, mqasim@aus.edu

Supplementary Note 1 – Line waist analogy between femtosecond Gaussian and Bessel beams under identical laser and scanning parameters

Table S1. Experimental parameters used to compare line waist for femtosecond Gaussian and Bessel beams.

Material	Pulse width	Repetition rate	Scan speed	Line spacing	Power	No. of scans	Input beams		Converter	Focusing lens
W	250 fs	50 kHz	10 mm/s	Single line	10 W	1	Bessel-Gauss	Gaussian	Axicon for Bessel-Gauss	5 cm convex lens

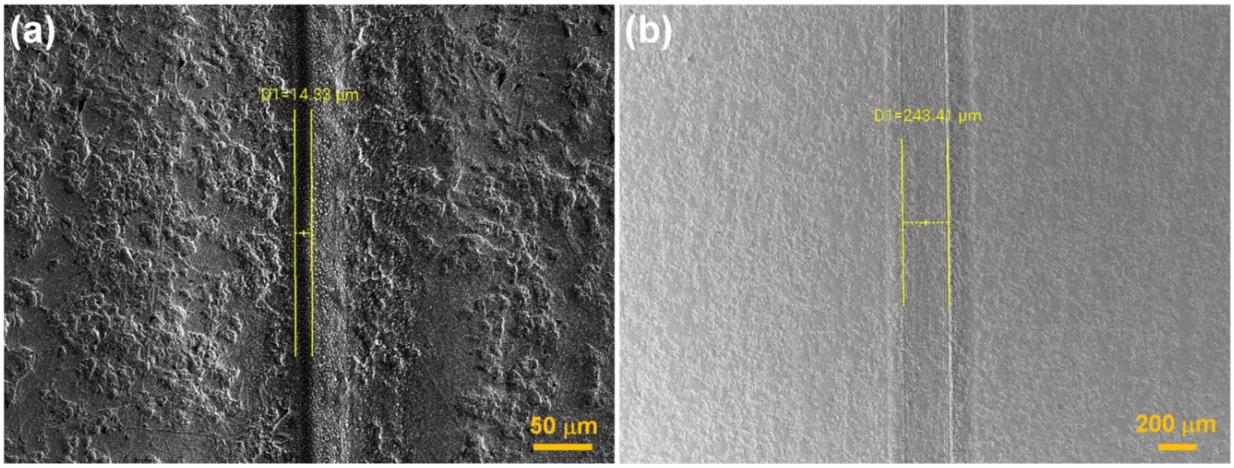

Figure S1. Single line inscribed on W with femtosecond (a) Bessel-Gauss and (b) Gaussian beams

As shown in the above SEM micrographs, the Bessel beam is much more focusable than Gaussian and for this reason, the Bessel bean could give a line almost 17 times thinner than Gaussian on W for the same scan conditions (Figure S1). Further, the neighborhood of the Bessel-inscribed line is relatively more occupied with ablation debris than the Gaussian-inscribed line.

Supplementary Note 2 – Surface morphology and cross-sectional analogy on femtosecond Gaussian and Bessel beams structured W

Table S2. Experimental parameters used for comparing surface morphology using femtosecond Gaussian and Bessel beams.

Material	Pulse width	Repetition rate	Scan speed	Line spacing	Power	No. of scans	Structuring beam		Converter	Focusing lens
W	250 fs	50 kHz	50 mm/s	40-100 µm	10 W	10	Bessel-Gauss	Gaussian	axicon	5 cm convex lens

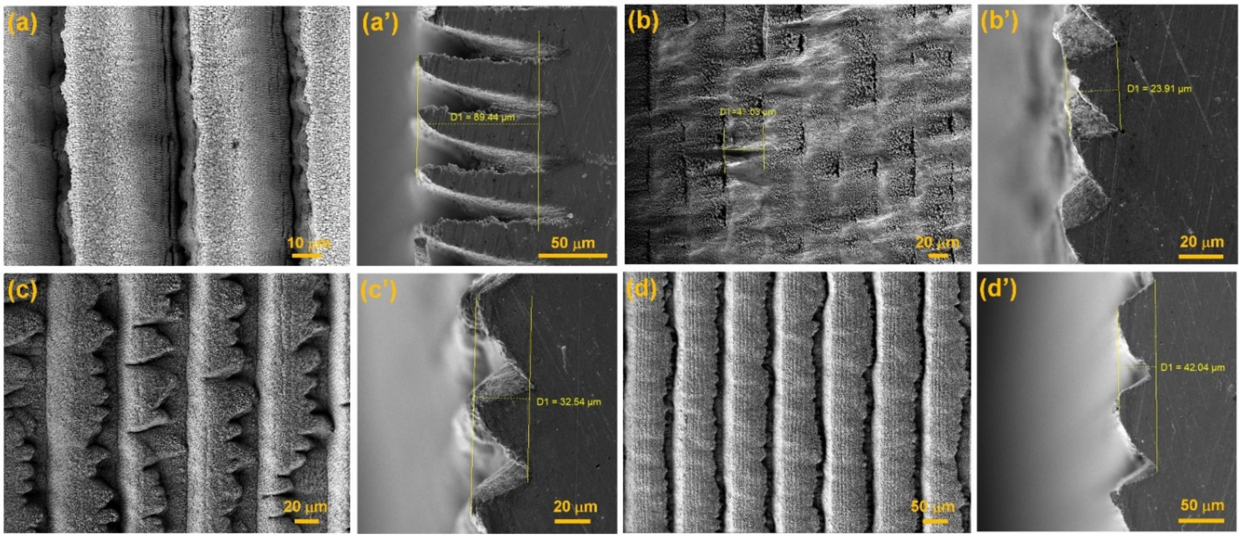

Figure S2. W structured with femtosecond (a) Bessel-Gauss beam with 40 μm line spacing, and (b-d) Gaussian beam with line spacing of (b) 40 μm, (c) 60 μm, and (d) 100 μm. (a'-d') are the corresponding cross-sectional micrographs of (a-d).

Supplementary Note 3 – EDS Patterns of femtosecond laser structured Al and Mo samples

EDS Patterns of femtosecond laser structured Al and Mo samples are given below. The Al:O and Mo:O at% ratios show O content lesser than stoichiometric for pure Al_2O_3 and MoO_3, respectively. This has to do with the femtosecond laser induced plasma, which ignites instantaneously and rapidly extinguishes to cause rapid cooling. There is not sufficient time for reactive O species in the plasma to react to stoichiometric amounts, rather substoichiometric oxides lacking strong long-range order, typically nanocrystalline or amorphous are produced.

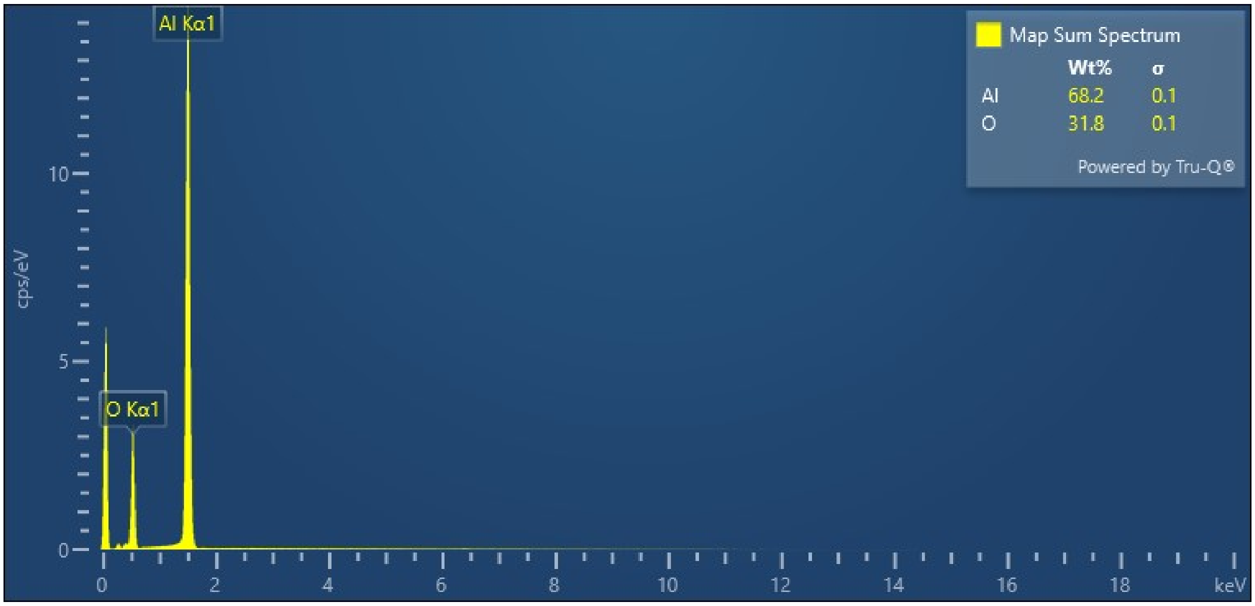

Figure S3. EDS pattern of Al structured with femtosecond Gaussian beam in air.

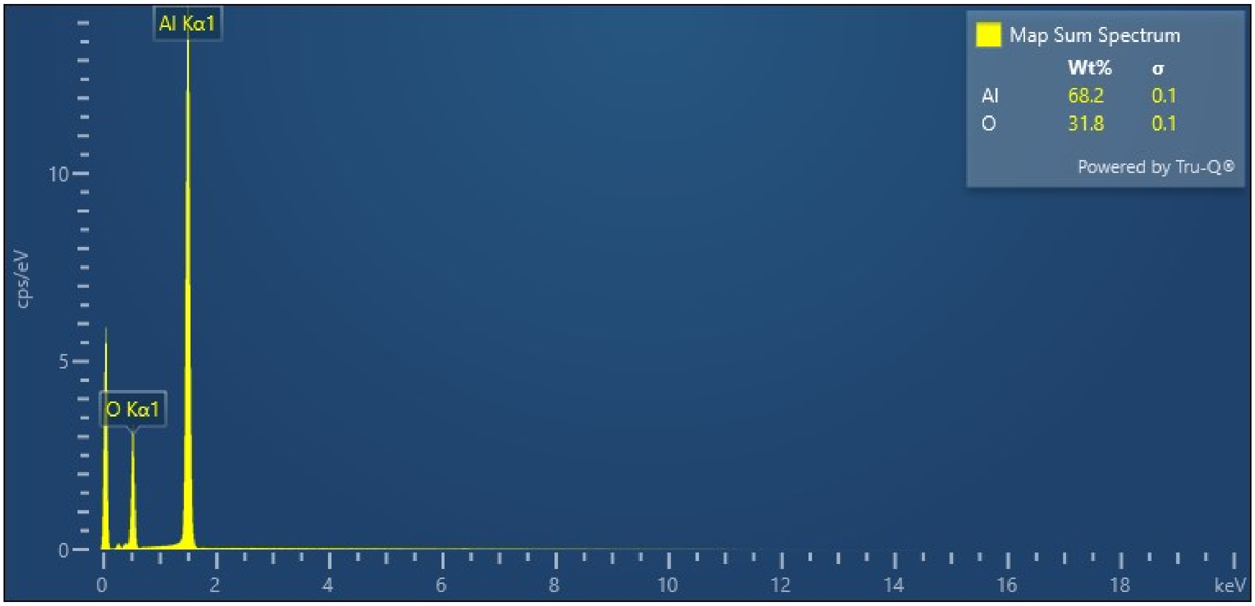

Figure S4. EDS pattern of Mo structured with femtosecond Gaussian beam in air.

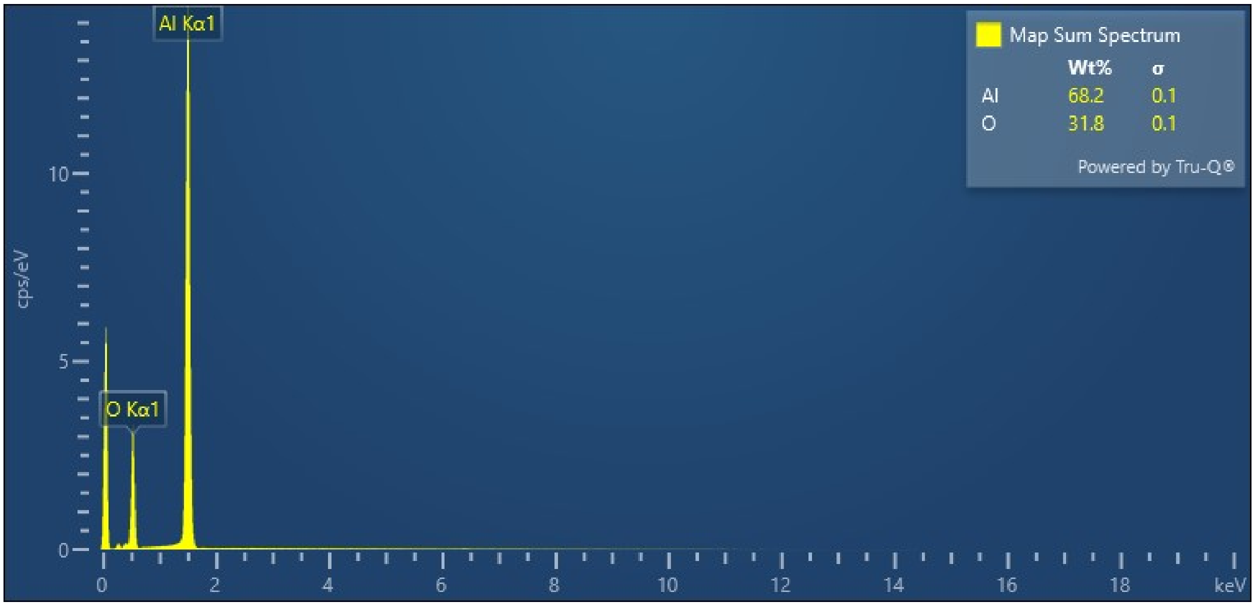

Figure S5. EDS pattern of Al structured with femtosecond Bessel-Gauss beam in air.

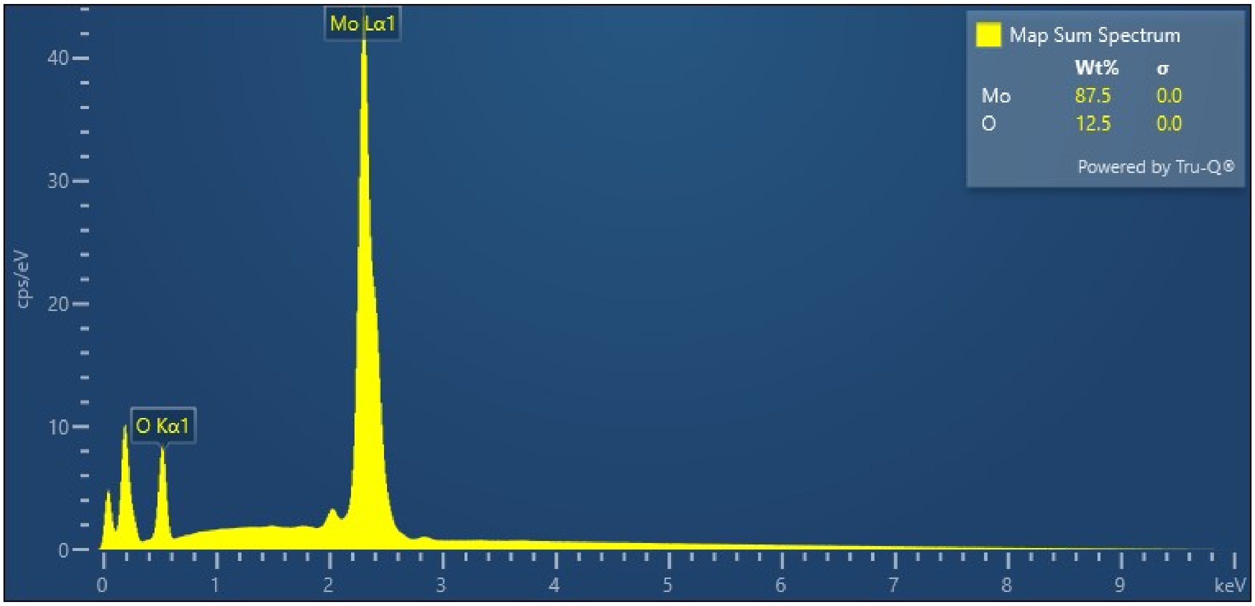

Figure S6. EDS pattern of Mo structured with femtosecond Bessel-Gauss beam in air.

Supplementary Note 4 – X-ray photoelectron spectroscopy survey spectra of AlO_x and MoO_x

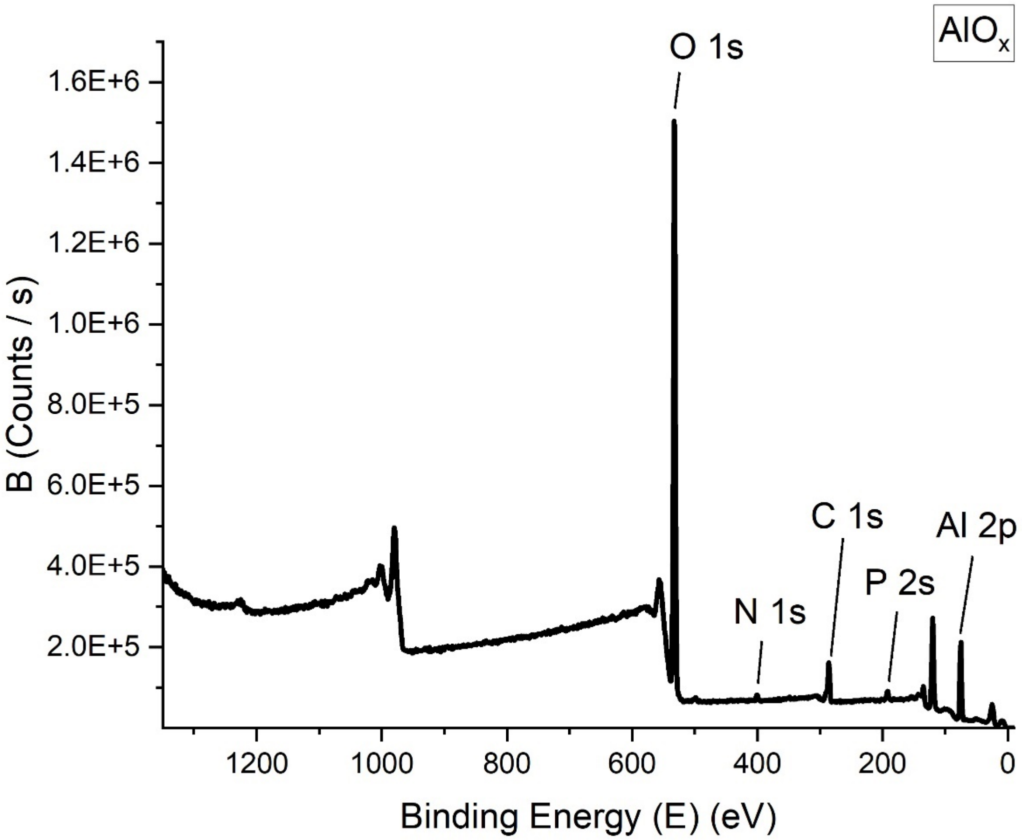

Figure S7. XPS survey spectrum of AlO_x produced with femtosecond laser pulses of Gaussian beam profile

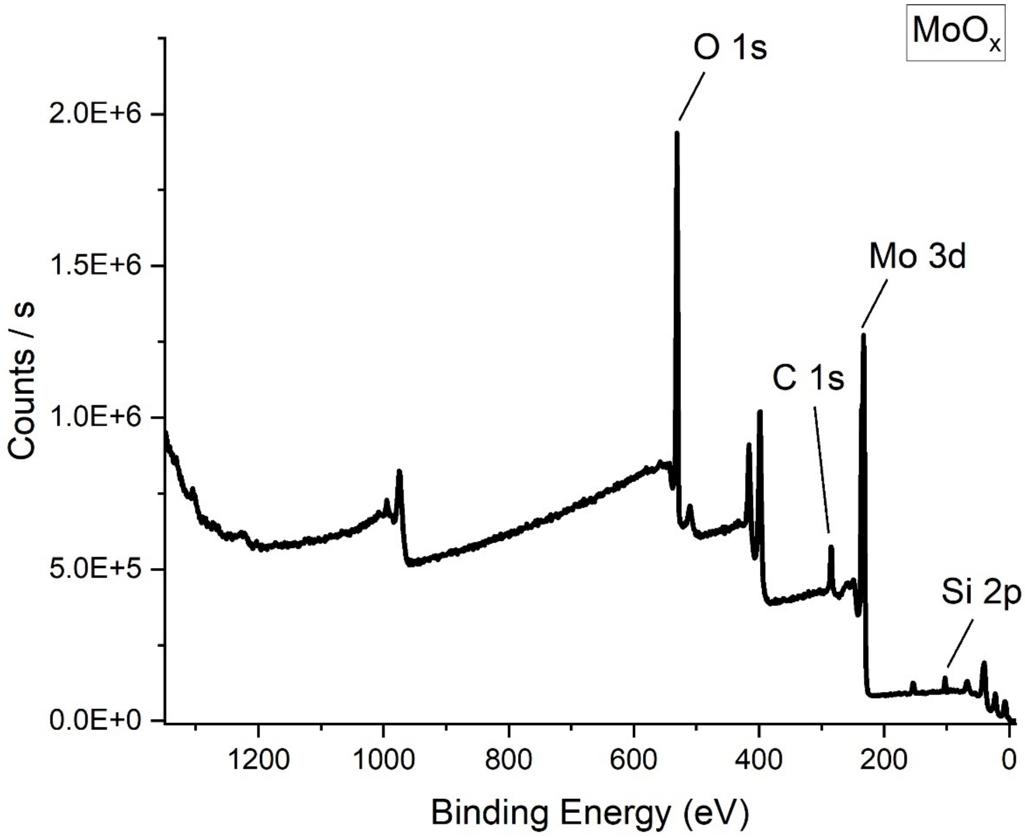

Figure S8. XPS survey spectrum of MoO_x produced with femtosecond laser pulses of Bessel beam profile

Supplementary Note 5 – IR analysis

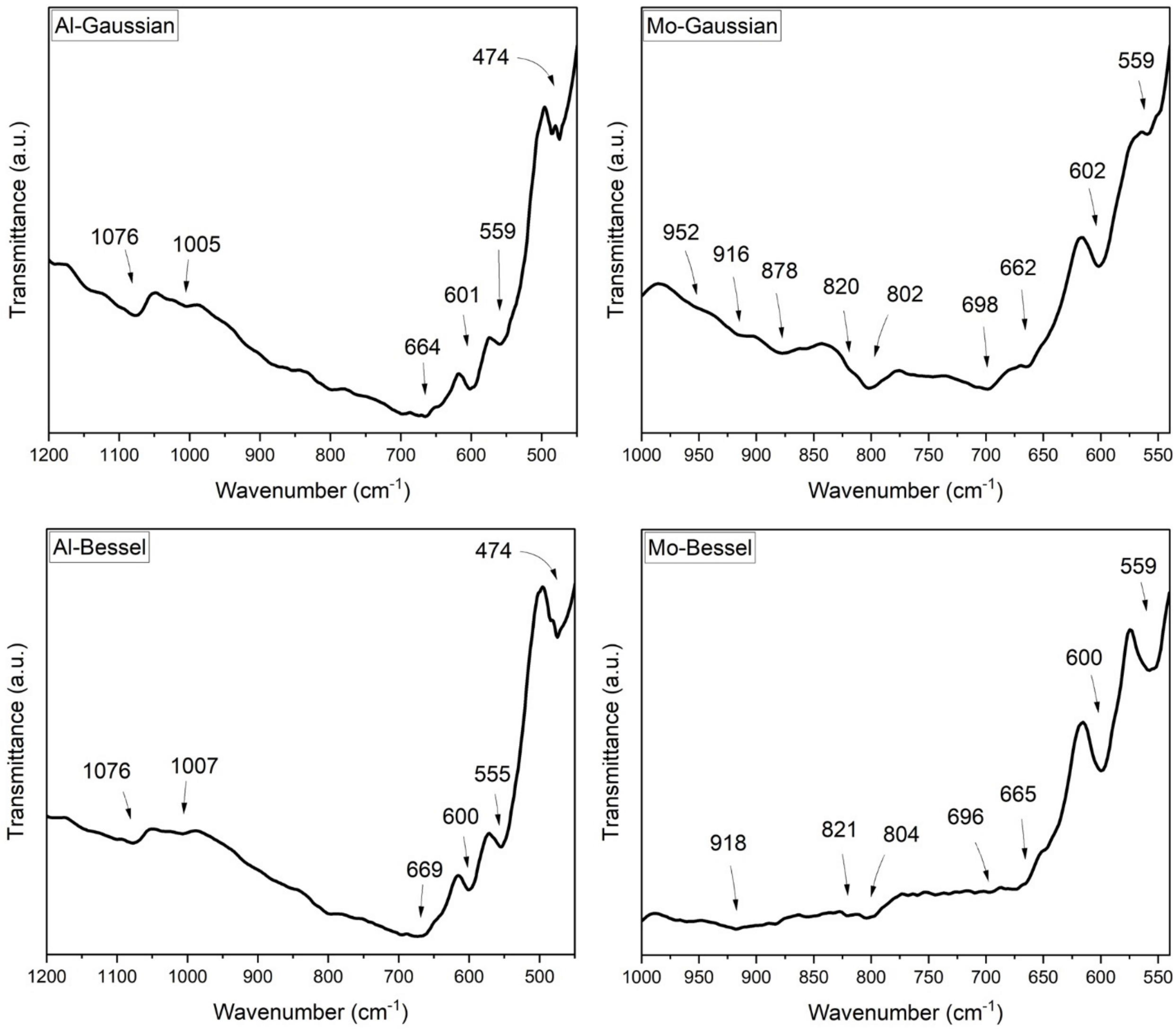

Figure S9. IR spectra of Al-Gaussian, Al-Bessel, Mo-Gaussian, and Mo-Bessel.

The FTIR spectra (**Figure S9**.) of Al-Gaussian and Al-Bessel confirm amorphous aluminum oxides and hydroxides present in these samples. The peaks in the 950-1100 cm^{-1} indicate Al-O-H, suggesting the presence of surface hydroxyls such as boehmite (AlOOH) and $Al(OH)_3$. Further, the absence of sharp peaks in the 700-850 cm^{-1} range suggests the presence of amorphous alumina. Moreover, the very broad peak in the 615-720 cm^{-1} is also indicative of amorphous Al_2O_3. Similarly, the peaks in the 450-500 cm-1 range also hint towards Al-O deformations in Al_2O_3 and boehmite. Lastly, the peaks in the 500-615 cm^{-1} indicate Al-O-Al bonds in alumina [1–4].

The IR spectra (**Figure S9**) of Mo-Gaussian and Mo-Bessel indicates mixed Mo oxide phases existing in both these samples. Since pure MoO_3 is signified by strong terminal Mo=O vibrations at 990 cm^{-1}, the presence of peaks between 900 and 990 cm^{-1} is indicative of either Mo^{6+} or substoichiometric oxides of mixed valence states (e.g., Mo^{5+}/Mo^{6+}). Further, the peaks in the 600–800 cm^{-1} range indicate bridging Mo–O–Mo bonds. Similarly, non-stoichiometric MoO_x (e.g., Mo_8O_{23}) is also represented in this range (e.g., 698

cm^{-1}). Likewise, the peaks at wavenumbers < cm^{-1} (e.g., 559 cm-1) signify Mo^{4+} (MoO_2).[5–7]. Though the intensities are somewhat different, the above peaks are present in both the Mo-Gaussian and Mo-Bessel.

Supplementary Note 6 – Raman shifts reported in literature

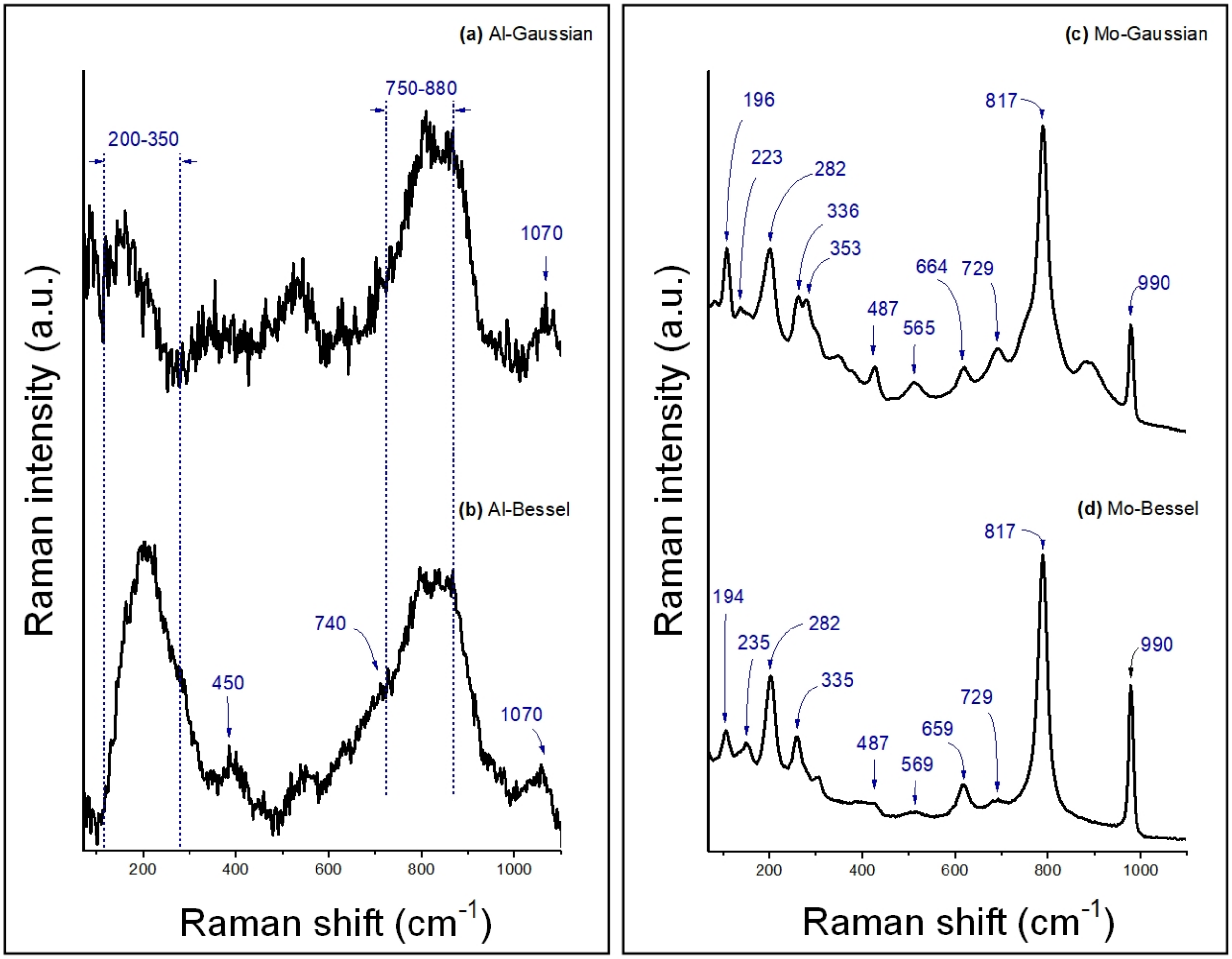

Figure S10. Raman spectra of (a, b) Al and (c, d) Mo structured in air with femtosecond (a, c) Gaussian and (b, d) Bessel beams.

The femtosecond laser treatment, which involves instantaneous ignition, followed by rapid cooling in ambient air, produces highly disordered alumina phases [8]. **Figure S10**(a) and (b) details Raman spectra of Al-Gaussian and Al-Bessel-Gauss solar absorbers, respectively. Such low intensity and broad peak patterns are typical of amorphous Al oxides [9]. Several Raman bands corresponding to Al–O vibrations were detected in the spectral range between 200 and 880 cm^{-1}. All observed Raman peaks are relatively weak and broad due to strong lattice disorder and O defects [1011]. We associate the strong lattice disorder with the high annealing temperatures and long dwelling times required to produce stable Al_2O_3 phases [12], compared to the short lifetime of the femtosecond laser-induced plasma. Based on previous reports, the broadband in the range of 200 – 350 cm^{-1} composed of three peaks around 250, 320, and 350 cm^{-1}, attributed to the bending mode of 4-coordinated AlO tetrahedron (AlO_4), deformations in tetrahedron and octahedron (6-coordinated AlO (AlO_6)) as well as bending modes of AlO_6 in α-Al_2O_3 phase [13]. The weak Raman peak at ~450 cm^{-1} was previously ascribed to AlO_6 stretching mode [14]. Previous studies on Al_2O_3 also reported

several unassigned bands located between 700 and 1000 [15]. However, the presence of a broad spectral signature in the mentioned range suggests the contribution of the AlO_4 stretching mode, whose position given by different authors is in the range of 750 – 880 cm^{-1}. Another band identified in the interrogated range appears at 740 cm^{-1} due to OH torsional mode [16]. Finally, the small signal at approximately 1070 cm^{-1} appears due to AlOH symmetric bending [8,10,17,18]. From the above result we infer that the surface of both Al-Gaussian and Al-Bessel-Gauss is primarily constituted by amorphous Al oxides (AlO_x).

Figure S*10*(c) and (d) are representative of the Raman spectra of Mo-Gaussian and Mo-Bessel-Gauss absorbers, respectively. Bands associated with different vibration modes of Mo oxides, such as MoO_2, MoO_x ($2<x<3$), and MoO_3, were detected in the 160 – 1000 cm^{-1} range in both Mo-Gaussian and Mo-Bessel-Gauss [19–22]. Contrary to the AlO_x, the Mo oxides are characterized by more intense peaks, signifying improved crystallinity. We attribute the enhanced crystallinity to the extended annealing time available during plasma cooldown [23]. This extended time is a result of the significantly lower annealing temperatures required to produce crystalline Mo oxides [24], coupled with the reduced scanning speed. Generally, the observed Raman signals in the Mo oxides are due to vibrational modes associated with MoO_4 tetrahedron [25] and MoO_6 octahedra [26]. MoO_2 is exclusively represented by the band around 569 cm^{-1} [20,21]. The bands for MoO_x ($2<x<3$) are identified around 196, 223, 235, 282, 335, 353, 487, 565, 659, 729, 817 and 990 cm^{-1} [20]. Similarly, the bands signifying the presence of MoO_3 are located around 336 and 664 cm^{-1} [20]. The band around 194 cm^{-1} may be assigned to both MoO_x and MoO_3. The abundance of bands discussed above, infers to the substoichiometric nature ($x<3$) of both the Gaussian and Bessel-Gauss samples. The vibration modes are further detailed in **Table S3**.

Table S3. Raman shifts (cm^{-1}) of Mo oxides [27]

MoO_2	MoO_x ($2<x<3$)	MoO_3	Assignment
	194, 196	194	A_g-$\delta(O_2Mo_2)_n$
	223,_235		A_g-$\delta(OMo_2)$
	282		B_{2g}-$\delta(O = Mo)$
	335	336	A_g-$\delta(OMo_3)$
	353		A_g-$\delta(O = Mo)$
569	487, 565, 728		m-MoO_2
	660	666	B_{3g}-$\nu(OMo_3)$
	816		B_{1g}-$\nu(OMo_2)$
	990		A_g, B_{1g}-$\nu(O = Mo)$

Supplementary Note 7 – X-ray photoelectron spectroscopy depth profiling of AlO_x and MoO_x evaporators

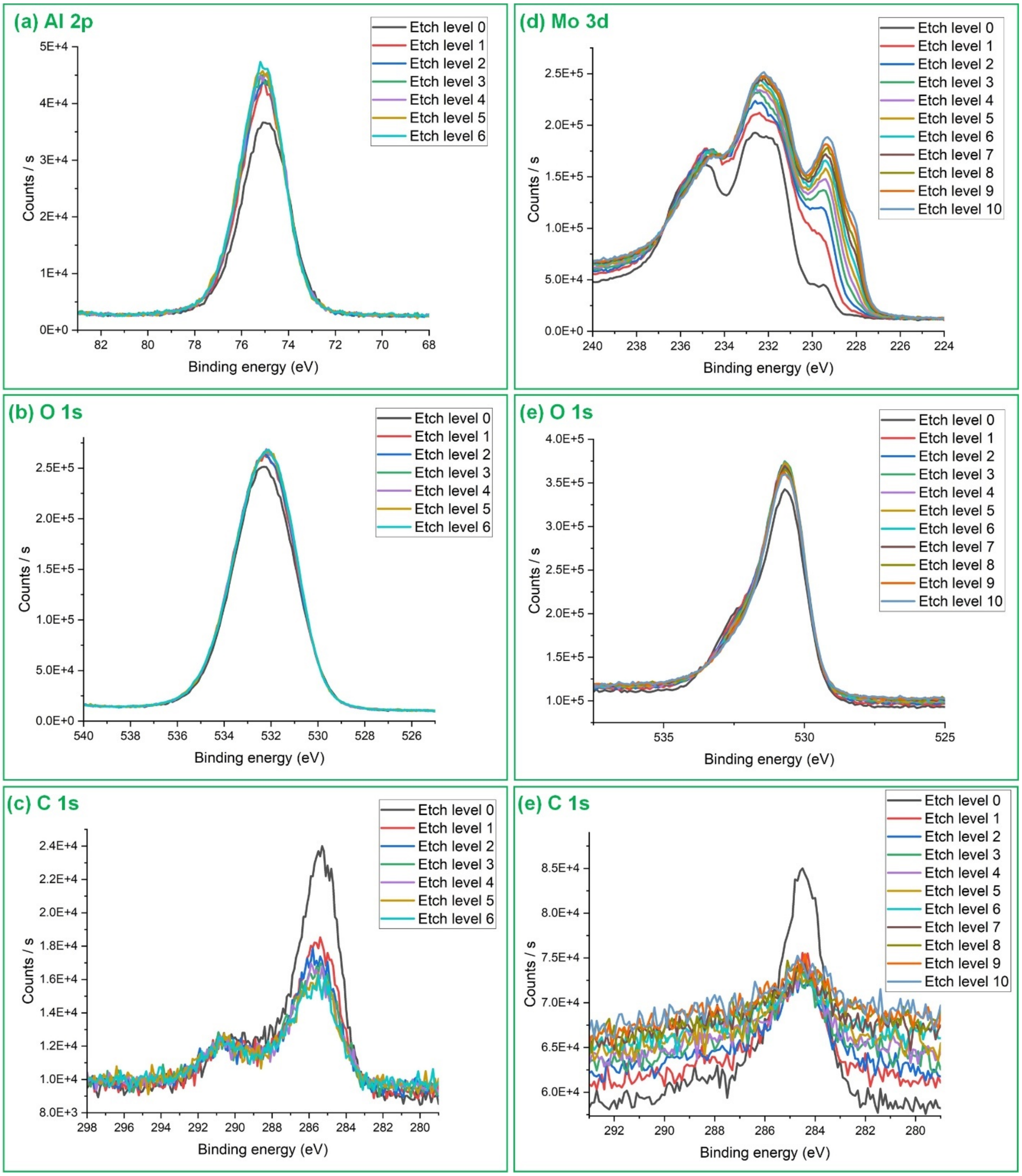

Figure S11. XPS depth profiling of (a-c) AlO_x and (d-f) MoO_x

Supplementary Note 8 – UV-Vis-NIR reflectance spectra of unstructured flat metals

Figure *S12* provides total spectral reflectance of the unstructured/bare samples. The reference in this case was a typical rough $BaSO_4$ surface. It can be seen that the metallic surfaces reflect more than the reference samples and therefore, the total reflectance is more than 100%. It can be observed that unstructured Al is more reflecting than unstructured Mo.

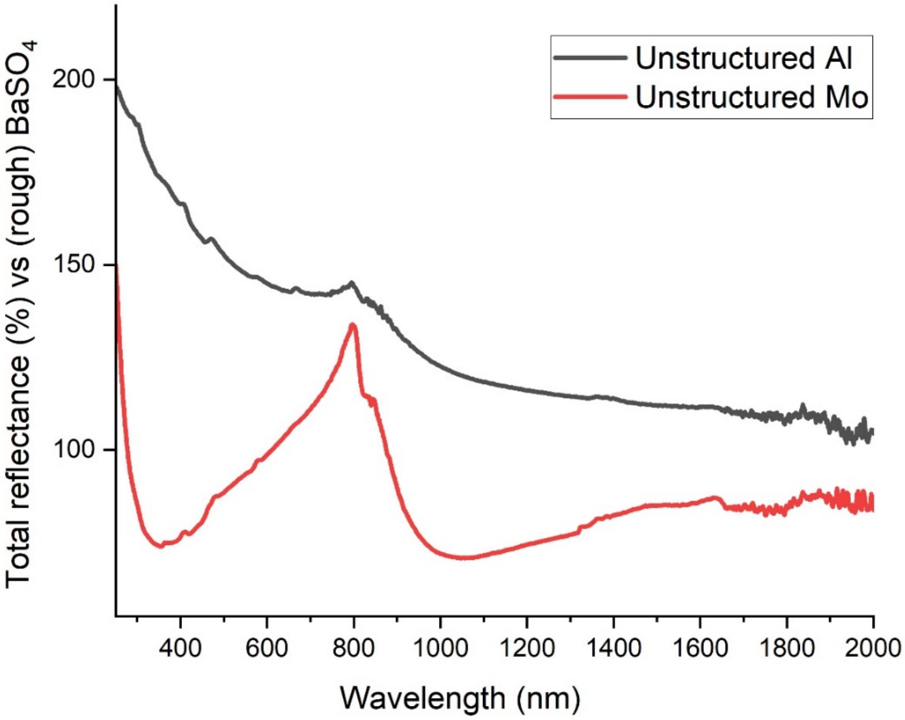

Figure *S*12. Total reflectance (%) from flat unstructured Al and Mo with reference to a highly diffuse reflecting $BaSO_4$.

Supplementary Note 9 – Temperatures attainable on the AlO_x and MoO_x evaporators

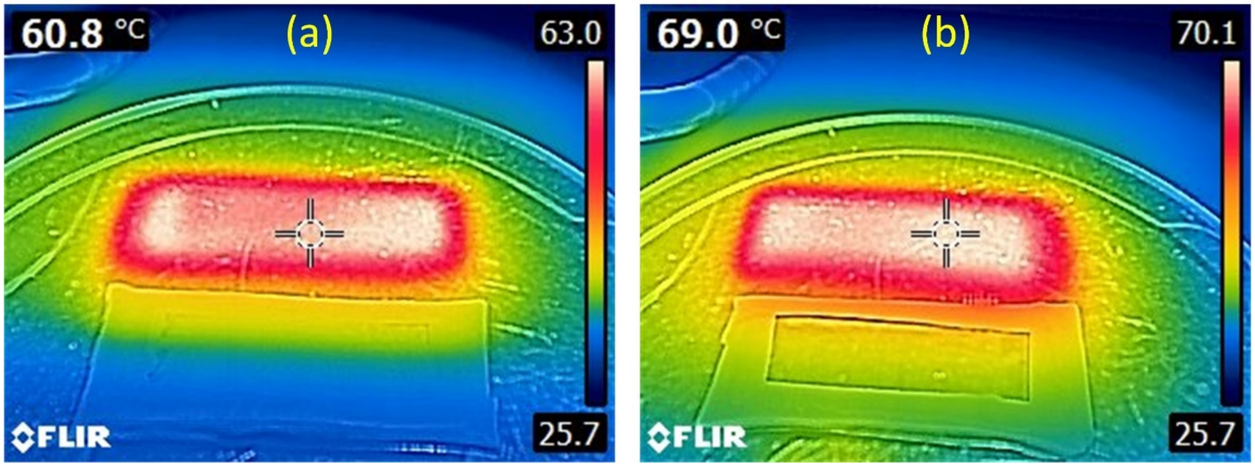

Figure *S*13. Dry surface temperature profiles of (a) AlO_x and (b) MoO_x due to photothermal heating under 1 Sun irradiance

Supplementary Note 10 – Estimation of surface area of water body confined in the microchannels

It has been known that the enhanced evaporation rate is not due to the increased surface area of the solar evaporators but instead due to lowering in the enthalpy of the liquid-vapor phase transition, H_{LV}, facilitating higher evaporation rate [28]. To estimate the surface area of the water body within the microchannels in contact with air relative to that of bulk water having flat surface, high magnification contact angle measurements were acquired with a drop shape analyzer (DSA100M, Kruss GmbH). **Figure *S14*** (a,b) shows sideview of dry (**Figure *S14*** (a)) and wicking (**Figure *S14*** (a)) AlO_x (i.e., Al-Gaussian) produced with Gaussian beam, whereas **Figure *S14*** (c,d) depicts sideview of dry MoO_x (i.e., Mo-Bessel) produced with the Bessel beam. The estimated meniscus length within the AlO_x microchannels is 82.5% of a flat-water surface of bulk water. Thus, the water confined in the AlO_x microchannels maintains 82.5% of the total flat surface area of bulk water in contact with air, provided the geometric surface area of the evaporator equals the surface area of the bulk water. Our estimation is slightly higher than previously reported surface area (76.5 %) of water confined within laser-inscribed AlO_x microchannels [28]. Applying the above procedure to the MoO_x evaporator, the estimated water surface area on MoO_x was found out to be ~85% of a flat water surface with equal geometric area (**Figure *S14*** (c,d)).

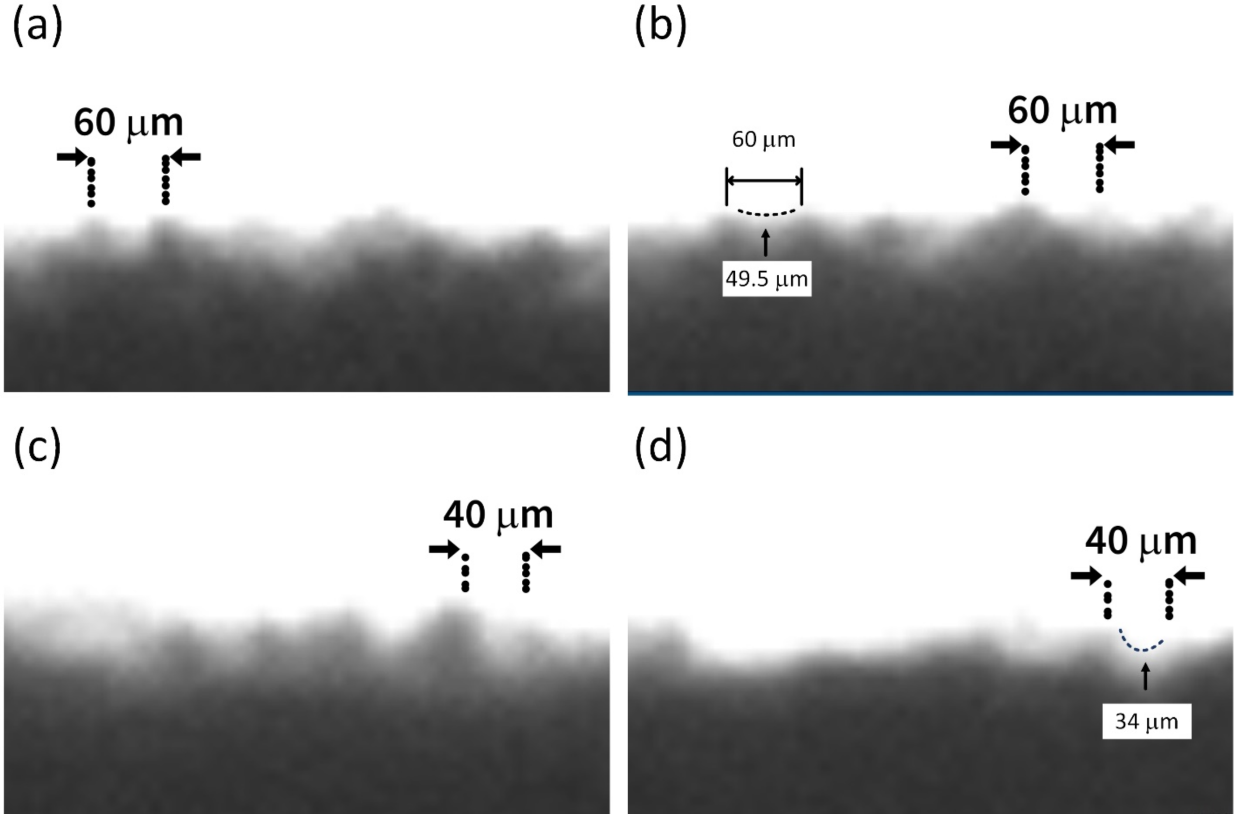

Figure *S*14. Side view of AlO_x and MoO_x evaporators (a), (c) without and (b), (d) with water wicking the microchannel arrays.

To further elucidate the water surface coverage during wicking 3.5 wt% NaCl solution (aq) vertically against gravity, metallurgical microscopy (**Figure *S15*** (a,b,d,e)) was done. **Figure *S15*** (a) and (d) show cross-sectional metallurgical microscopy images of the respective AlO_x and MoO_x before wicking, whereas **Figure *S15*** (b) and (d) are the corresponding cross-sectional images after wicking. **Figure *S15*** (c) and (f) are the respective camera captured photographs of AlO_x and MoO_x after wicking. The SEM microscopy images of dry MoO_x are shown in **Figure *S15*** (g) and (h), whereas the corresponding camera captured photograph of the dry MoO_x is depicted in **Figure *S15*** (i). The capillary channels of AlO_x turned reddish compared to the orange-red appearance before wicking (noting that focusing doesn't alter the colors). The sections which have been wetted mostly turned reddish. It reveals that there exists a strong adhesion between the saline water and the AlO_x such that the microchannels are almost entirely wetted. On comparing the dry and the wet AlO_x samples, it is observed that the channel depths appear more blur compared to the walls on the wet sample, thus indicating the water body confined within the channels. This is in confirmation with our above findings inferring 82.5% water surface area confined within the microchannels compared to a flat-water body of equivalent geometric surface area. A glimpse of this wicking property of AlO_x microchannels is shown in **Figure *S15*** (i).

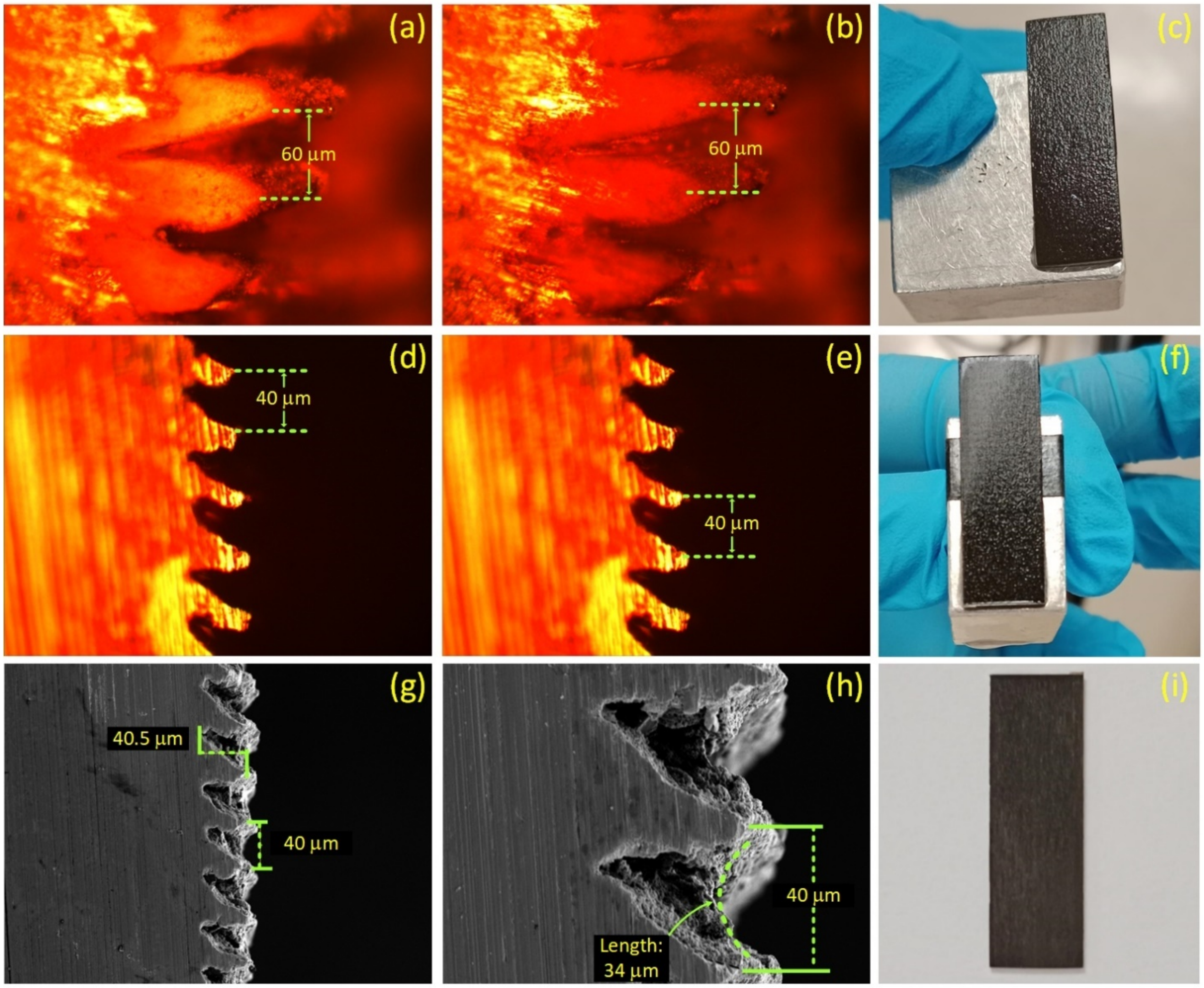

Figure *S*15. Metallurgical microscope cross-sectional images of AlO_x (a) without and (b) with wicking. (c) is a camera captured photograph of AlO_x after wicking against gravity. Metallurgical microscope images of MoO_x (d) without and (e) with wicking. (f) is a camera photograph of MoO_x after wicking against gravity. (g) and (h) are low and relatively high magnification SEM image of dry MoO_x, respectively, depicting nanochannels filling a fraction of the microchannels. (i) is the corresponding camera captured photograph of dry MoO_x evaporator.

Contrary to the AlO_x microchannels, which depict a strong color shift upon wetting, the metallurgical microscope images of MoO_x do not show any considerable shift in color upon wetting (**Figure *S15*** (d) vs **Figure *S15*** (e)), though the sample is highly wicking (**Figure *S15*** (f)). As shown in **Figure *S15*** (g) and (h), the MoO_x nanochannels, which superimpose the microchannels, occupy a considerable fraction of the total microchannel volume. A thin layer of the saline water is believed to be wicking these hierarchical features and is believed to be the reason for sustaining the dry color even after wetting in metallurgical microscopy. Considering, the nanochannels within the microchannels as completely soaked, the area of the water body in contact with air within the MoO_x microchannels then equals the geometric surface area of the nanochannel structures. This fact is highlighted in **Figure *S15***, suggesting ~85% surface coverage compared to a flat-water body of geometric area equal to that of the MoO_x evaporator. Roughly the same amount of coverage was deduced from the above water contact angle measurements as well (**Figure *S*14** (c) and (d)).

Supplementary Note 11 – Contributions of different hydrogen bonding networks

The following **Figure *S16*** details the observed hydrogen bonding states from deconvolution of OH stretching vibrations for water in the bulk and close to the absorber interface.

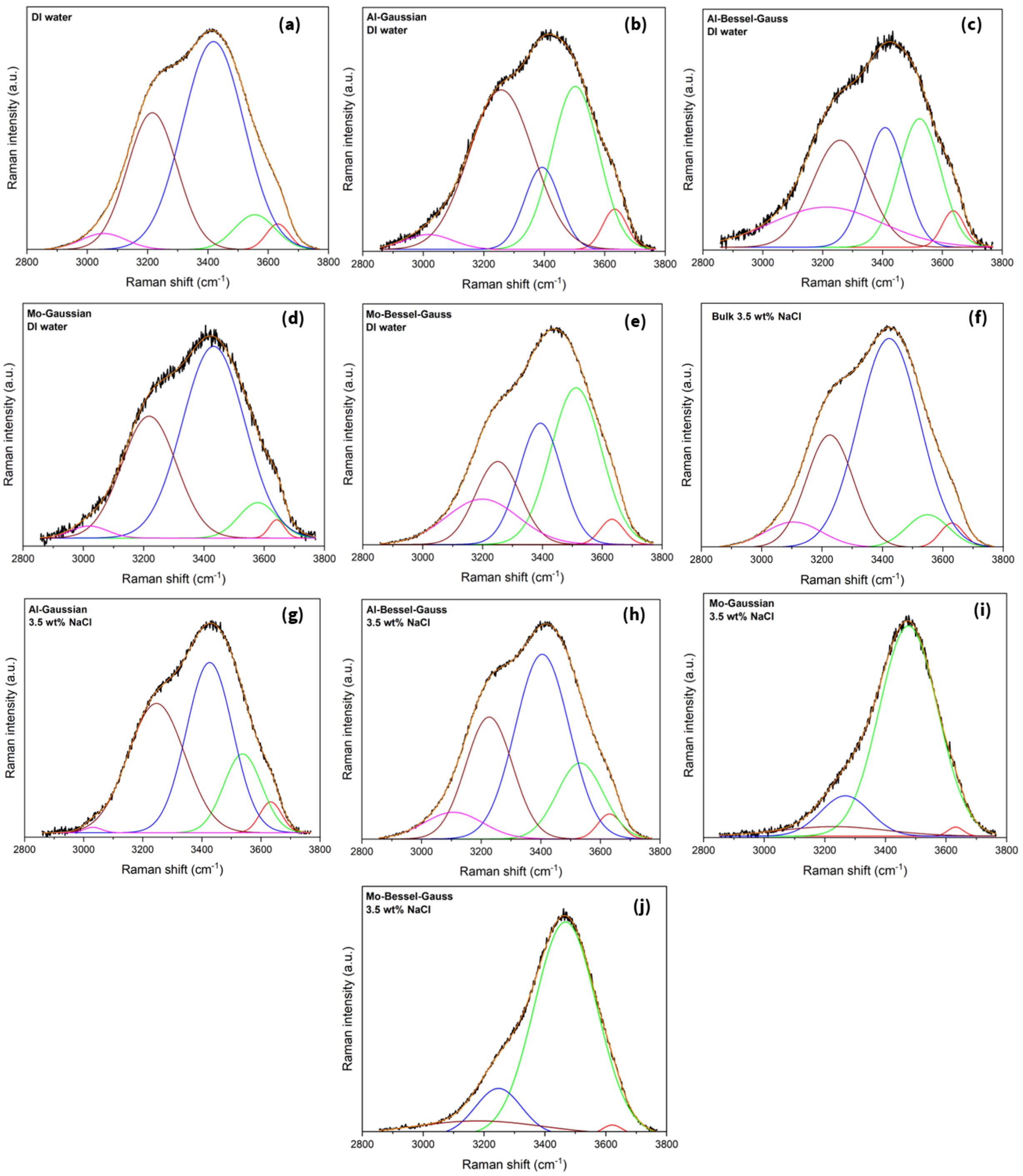

Figure *S*16. Deconvoluted OH stretching vibration in the Raman spectra.

The **Table S4** given below provides the percentage contributions from different types of hydrogen bonding in the bulk and on the surface of the solar absorbers. Values have been calculated based on the sub-peak integral area after deconvolution of the OH stretching vibration in the Raman spectra into five Gaussian sub-peaks.

Table S4. Percentage contributions of different hydrogen bonding types calculated by deconvolution of Raman spectra.

	**Free-OH (%)**	**DDA-OH (%)**	**DA-OH (%)**	**DDAA-OH (%)**	**DAA-OH (%)**
	3640 cm^{-1}	3573 cm^{-1}	3434 cm^{-1}	3226 cm^{-1}	3005 cm^{-1}
			**DI water**		
Bulk	2.7	6.4	57.3	30.4	3.2
Al-Gaussian	4.3	34.0	12.8	45.7	3.2
Al-Bessel-Gauss	3.8	25.2	22.3	27.8	20.8
Mo-Gaussian	1.3	6.6	57.7	32.2	2.2
Mo-Bessel-Gauss	2.9	38.4	25.1	17.9	15.8
			**3.5 wt% NaCl solution**		
Bulk	2.7	6.4	59.7	24.5	6.7
Al-Gaussian	3.5	15.8	41.6	38.6	0.5
Al-Bessel-Gauss	2.8	16.5	46.6	26.8	7.3
Mo-Gaussian	0.9	79.6	12.9	6.5	0
Mo-Bessel-Gauss	1.1	76.3	12.2	10.4	0

Supplementary Note 12 – Boiling point elevation

The boiling point elevation (BPE), being a colligative property, is dependent on the concentration of the non-volatile in water. When foreign ions such as Na^+ and Cl^- are introduced into water, water molecules close by these ions are attracted towards the ions with the water dipoles orient to balance the ion charge. Due to the ion-dipole attractions, the ions become hydrated by a certain number of water molecules, called the primary waters of hydration. The number of water molecules responsible for the primary waters of hydration depend on the specific ion in interaction. The primary water of hydration in the bulk water is 4 and 1 for Na^+ and Cl^-, respectively. To balance this localized charge emerging due to the ordered water dipoles surrounding the ion, a secondary layer of partially ordered water molecules emerges outside this layer. This redistribution of interactions is responsible for increasing the surface tension and boiling point of sea water compared to pure water [29].
The BPE results in a reduction of the evaporation rate of the bulk water. The BPE is a consequence of the extra temperature and extra energy required to equal the vapor pressure to that of the ambient. Salinity, solvent temperature, and external pressure influence BPE. Increasing the solute concentration promotes further BPE because of the increasing solute-solvent interactions at higher concentrations. Several correlations have been proposed to calculate BPE as a function of salt concentration and temperature [30]. Below we discuss one such correlation method.

BPE can be correlated with the weight percent ($wt\%$, $1 \leq wt\% \leq 16\%$) of NaCl and the temperature (T in ℃, $10 \leq T \leq 160℃$) by the following relation [31].

$$BPE = A(wt\%) + B(wt\%)^2 + C(wt\%)^3$$

Here
$$A = 8.325 \times 10^{-2} + 1.883 \times 10^{-4}T + 4.02 \times 10^{-6}T^2$$

$B = -7.625 \times 10^{-4} + 9.02 \times 10^{-5} T - 5.2 \times 10^{-7} T^2$

$C = 1.522 \times 10^{-4} - 3 \times 10^{-6} T - 3 \times 10^{-8} T^2$

As per the above expression, the BPE equals ~0.33 ℃ for 3.5 wt% NaCl(aq) at 25 ℃.

Supplementary Note 13 – Open circuit potential (OCP) measurement in DI water

Other than increasing the boiling point of water, NaCl addition is also influencing the development of a charged interface between the absorber and the electrolyte. An electrical double layer (EDL), which is an array of charged species, develops at the absorber/electrolyte interface. The EDL comprises of a thin uniform monolayer of adsorbed ions cum water dipoles covering the electrode surface. The charge imbalance due to the adsorbed monolayer is countered by unabsorbed hydrated counterions of opposite polarity, which arrange themselves next to the adsorbed monolayer in the solution [29,32].

All ions, whether anions or cations can specifically adsorb on the electrode in specific charge regions. Ions with a proper hydration sheath have lower tendencies to specifically adsorb on unbiased surfaces than ions with lesser hydration. Specific adsorption is more common for larger size ions with less primary hydration. Ions of radii greater than 1.4 Å can adsorb much easily on surfaces [32], and that is why Cl^- (ionic radius of 1.64 Å) can adsorb more than Na^+ (ionic radius of 1.17 Å) [33]. These electrolyte ions compete with water to adsorb on the solid surface [34]. Due to EDL, a potential difference appears across the electrode/solution interface.

The rest potential is therefore the result of the cumulative adsorption interactions at the interface between the absorber and the electrolyte. The rest potentials for AlO_x and MoO_x in DI water are given in **Figure S17**. AlO_x depicts a rest potential of ~-0.35 V (vs Ag/AgCl (3M KCl)), whereas MoO_x has a rest potential of ~-0.11V.

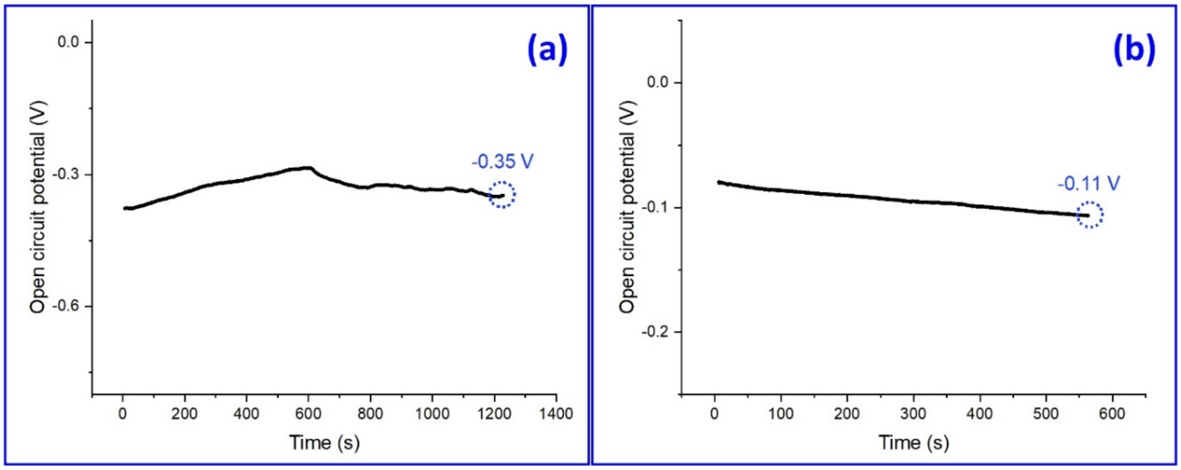

Figure S17. Chronopotentiograms to measure the OCP of (a) AlO_x and (b) MoO_x in DI water.

References

1 W. Daniell, U. Schubert, R. Glöckler, A. Meyer, K. Noweck and H. Knözinger, *Appl Catal A Gen*, 2000, **196**, 247–260.

2 V. Sanchez Escribano, G. Garbarino, E. Finocchio and G. Busca, *Top Catal*, 2017, **60**, 1554–1564.

3 G. Busca, *Catal Today*, 2014, **226**, 2–13.

4 M. Trueba and S. P. Trasatti, *Eur J Inorg Chem*, 2005, **2005**, 3393–3403.

5 M. A. Hosseini and M. Ranjbar, *Plasmonics*, 2018, **13**, 1897–1906.

6 A. Kumar, Vandana, M. Dutta, S. K. Srivastava and P. Pathi, *Journal of Materials Science: Materials in Electronics*, 2024, **35**, 1–13.

7 N. Maheswari and G. Muralidharan, *Appl Surf Sci*, 2017, **416**, 461–469.

8 I. González de Arrieta, A. Zaki, A. Canizarès, E. Véron, C. Genevois, L. del Campo, C. Blanchard and O. Rozenbaum, *Spectrochim Acta A Mol Biomol Spectrosc*, 2023, **298**, 122795.

9 Z. Wang, B. Sun, H. Ye, Z. Liu, G. Liao and T. Shi, *Appl Surf Sci*, 2021, **546**, 149094.

10 M. Baronskiy, A. Rastorguev, A. Zhuzhgov, A. Kostyukov, O. Krivoruchko and V. Snytnikov, *Opt Mater (Amst)*, 2016, **53**, 87–93.

11 M. V. Shugaev, C. Wu, O. Armbruster, A. Naghilou, N. Brouwer, D. S. Ivanov, T. J. Y. Derrien, N. M. Bulgakova, W. Kautek, B. Rethfeld and L. V. Zhigilei, *MRS Bull*, 2016, **41**, 960–968.

12 Y. O. Leonova, M. A. Sevostyanov, D. O. Mezentsev, D. R. Khayrutdinova and A. S. Lysenkov, in *Journal of Physics: Conference Series*, IOP Publishing, 2021, vol. 1942, p. 012052.

13 A. Boumaza, L. Favaro, J. Lédion, G. Sattonnay, J. B. Brubach, P. Berthet, A. M. Huntz, P. Roy and R. Tétot, *J Solid State Chem*, 2009, **182**, 1171–1176.

14 A. S. Barker, *Physical Review*, 1963, **132**, 1474.

15 A. S. Barker, *Physical Review*, 1963, **132**, 1474.

16 M. C. Stegmann, D. Vivien and C. Mazieres, *Spectrochim Acta A*, 1973, **29**, 1653–1663.

17 T. Dash, T. K. Rout, B. B. Palei, S. Bajpai, S. Kundu, A. N. Bhagat, B. K. Satpathy, S. K. Biswal, A. Rajput, A. K. Sahu and S. K. Biswal, *SN Appl Sci*, 2020, **2**, 1147.

18 P. A. Pajaczkowska, P. Reiche, D. Klimm and G. Majumdar, in *Reference Module in Materials Science and Materials Engineering*, Elsevier, 2017.

19 B. Chae, Y. M. Jung, X. Wu and S. Bin Kim, *Journal of Raman Spectroscopy*, 2003, **34**, 451–458.

20 M. A. Camacho-López, L. Escobar-Alarcón, M. Picquart, R. Arroyo, G. Córdoba and E. Haro-Poniatowski, *Opt Mater (Amst)*, 2011, **33**, 480–484.

21 H. K. Puppala, A. T. Pelton and R. A. Mayanovic, *MRS Adv*, 2016, **1**, 2585–2590.

22 L. Aleksandrov, T. Komatsu, R. Iordanova and Y. Dimitriev, *Opt Mater (Amst)*, 2011, **33**, 839–845.

23 V. N. Rai and S. N. Thakur, *Laser-Induced Breakdown Spectroscopy, Second Edition*, 2020, 71–106.

24 M. Mattinen, P. J. King, L. Khriachtchev, M. J. Heikkilä, B. Fleming, S. Rushworth, K. Mizohata, K. Meinander, J. Räisänen, M. Ritala and M. Leskelä, *Mater Today Chem*, 2018, **9**, 17–27.

25 J. Bhagwan and J. I. Han, *Surfaces and Interfaces*, 2023, **36**, 102605.

26 H. Dai, X. Deng, A. Zhang, Y. Zhu, X. Xiao, Y. Wang and C. Zhou, *Int J Hydrogen Energy*, 2024, **68**, 1–7.

27 M. A. Camacho-López, L. Escobar-Alarcón, M. Picquart, R. Arroyo, G. Córdoba and E. Haro-Poniatowski, *Opt Mater (Amst)*, 2011, **33**, 480–484.

28 S. C. Singh, M. ElKabbash, Z. Li, X. Li, B. Regmi, M. Madsen, S. A. Jalil, Z. Zhan, J. Zhang and C. Guo, *Nature Sustainability 2020 3:11*, 2020, **3**, 938–946.

29 E. McCafferty, *New York*.

30 N. A. A. Qasem, M. M. Generous, B. A. Qureshi and S. M. Zubair, *Springer Water*, 2023, **Part F1299**, 155–172.

31 H. El-Dessouky and H. Ettouney, *Fundamentals of salt water desalination*, 2002.

32 J. O. BOCKRIS, M. A. V DEVANATHAN and K. MÜLLER, in *Electrochemistry*, eds. J. A. FRIEND and F. GUTMANN, Pergamon, 1965, pp. 832–863.

33 K. S. Chua, *Nature 1968 220:5174*, 1968, **220**, 1317–1319.

34 T. Patniboon and H. A. Hansen, *Chemical Physics Reviews*, DOI:10.1063/5.0125654.
